# Can a Small Change in the Heterocyclic Substituent Significantly Impact the Physicochemical and Biological Properties of (*Z*)-2-(5-Benzylidene-4-oxo-2-thioxothiazolidin-3-yl)acetic Acid Derivatives?

**DOI:** 10.3390/s24051524

**Published:** 2024-02-27

**Authors:** Agata Szlapa-Kula, Slawomir Kula, Łukasz Kaźmierski, Anna Biernasiuk, Przemysław Krawczyk

**Affiliations:** 1 Institute of Chemistry, Faculty of Science and Technology, University of Silesia in Katowice, Szkolna 9 St., 40-007 Katowice, Poland; 2 Urology and Andrology, Department of Tissue Engineering, Collegium Medicum, Nicolaus Copernicus University, M. Curie Skłodowskiej 9, 85-094 Bydgoszcz, Poland; lukasz.kazmierski@cm.umk.pl; 3 Department of Pharmaceutical Microbiology, Faculty of Pharmacy, Medical University of Lublin, 20-093 Lublin, Poland; anna.biernasiuk@umlub.pl; 4 Department of Physical Chemistry, Faculty of Pharmacy, Collegium Medicum, Nicolaus Copernicus University, Kurpińskiego 5, 85-950 Bydgoszcz, Poland; przemekk@cm.umk.pl

**Keywords:** rhodanine-3-acetic acid, Knoevenagel condensation, fluorescent probes, bioimaging, antimicrobial and antifungal activity

## Abstract

Rhodanine-3-acetic acid derivatives are attractive compounds with versatile effects. What is very important is that compounds of this type have many biological properties. They are tested, among others, as fluorescent probes for bioimaging and aldose reductase inhibitors. Rhodanine-3-acetic acid derivatives also have antibacterial, antifungal and anticancer activity. The presented work demonstrates that a slight change in the five-membered heterocyclic substituent significantly affects the properties of the compounds under consideration. Three rhodanine-3-acetic acid derivatives (**A-1**–**A-3**) were obtained in the Knoevenagel condensation reaction with good yields, ranging from 54% to 71%. High thermal stability of the tested compounds was also demonstrated above 240 °C. The absorption and emission maxima in polar and non-polar solvents were determined. Then, the possibility of using the considered derivatives for fluorescence bioimaging was checked. Compounds **A-1** and **A-2** were successfully used as fluorescent dyes of fixed cells of mammalian origin. In addition, biological activity tests against bacteria and fungi were carried out. Our results showed that **A-1** and **A-2** showed the most excellent antimicrobial activity among the newly synthesized compounds, especially against Gram-positive bacteria.

## 1. Introduction

The search for new chemical compounds with precisely defined physicochemical and biological properties has been a fundamental goal of scientific research for many years. This is primarily due to the growing requirements placed on chemical compounds used, among others, in technology, pharmacy and medicine. In recent years, derivatives of rhodanine-3-acetic acid have become increasingly popular in scientific research [1,2,3,4,5,6,7,8,9,10,11,12,13,14,15,16,17,18,19,20,21,22,23,24,25,26]. These compounds exhibit many interesting physicochemical and biological properties, so they are being investigated for numerous applications [1,2,3,4,5,6,7,8,9,10,11,12,13,14,15,16,17,18,19,20,21,22,23,24,25,26]. One of them is in dye-sensitized solar cells (DSSCs) in which derivatives of rhodanine-3-acetic acid can act as dyes [4,5,6,7,9,10,11,12,16,25]. This is possible because the mentioned group of compounds very often has a donor–acceptor (D-A) character. The heteroaromatic ring of rhodanine-3-acetic acid is an excellent acceptor group. The substituent (in position 5) acts as a donor. Due to their physicochemical properties, the derivatives of rhodanine-3-acetic acid have also been tested as fluorescent sensors for the selective detection of Ag^+^, Hg^2+^ and Pb^2+^ ions [14] and as fluorescent probes for bioimaging [3,15]. Moreover, the mentioned compounds can also be used as aldose reductase inhibitors [18]. A perfect example of this type is Epalrestat [18]. Rhodanine-3-acetic acid derivatives also have antibacterial and antifungal activity [1,2,8,13,17,20,21,22,23,24,26]. In the case of the antibacterial effect, it focuses mainly on Gram-positive bacteria [1,2,8,13,20,21,22,23,24,26]. The anticancer activity of the derivatives in question also deserves special attention [8]. 

This study aims to assess the influence of the heterocyclic substituent (pyrrolidine—**A-1**, pyrrole—**A-2** and imidazoles—**A-3**) on the physicochemical and biological properties of rhodanine-3-acetic acid derivatives, precisely three derivatives of 2-(5- benzylidene-4-oxo-2-thioxothiazolidin-3-yl)acetic acid (**A-1**–**A-3**). The considered substituents (pyrrolidine, pyrrole and imidazole) were selected so that their character changed from aliphatic to aromatic. Such minor structural changes made it possible to determine the relationship between the structure of the presented compounds and their properties. This also made it possible to trace the physicochemical and biological properties of the considered compounds in the context of the (aliphatic or aromatic) nature of the substituent. In the case of derivatives with aromatic substituents (pyrroles and imidazoles), the impact of an additional nitrogen atom on the considered properties was also assessed. All compounds were obtained by Knoevenagel condensation. Their structure was confirmed by NMR spectroscopic methods (^1^H, ^1^H-^1^H COSY, ^13^C, ^1^H-^13^C HMQC and ^1^H-^13^C HMBC). Then, optical tests were carried out for **A-1**–**A-3**. Based on the optical test results, the synthesized compounds (**A-1**–**A-3**) were tested as fluorescent dyes for imaging fixed and live cells. Moreover, the obtained derivatives were tested for antibacterial activity (against eight reference strains of Gram-positive bacteria and five strains of Gram-negative bacteria) and antifungal activity (against five strains of *Candida* spp. yeasts). Selected experimental data were confirmed by quantum-chemical calculations using density functional theory (DFT).

## 2. Materials and Methods

All data about materials, methods and experiments are located in the Supplementary Materials [27,28,29,30,31,32]. In addition, all spectra and analysis results are included in the Supplementary Materials [33,34,35,36,37,38,39,40,41,42,43,44,45,46,47,48,49,50,51,52,53,54].

## 3. Results and Discussion

### 3.1. Preparation and Characterization

**Figure 1:**
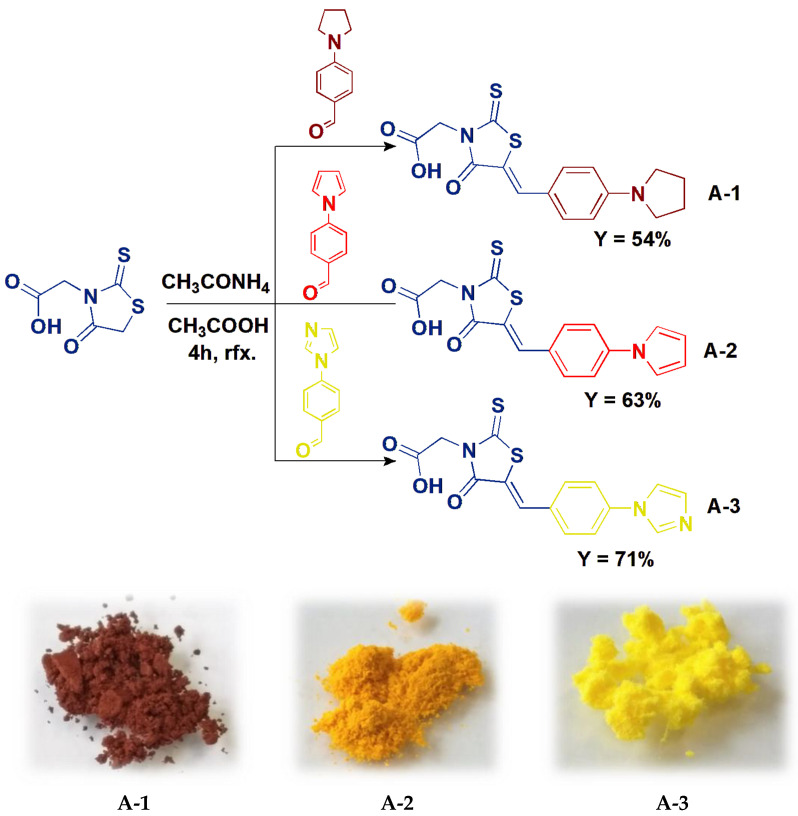
Synthesis of *(Z)-*2-(5-benzylidene-4-oxo-2-thioxothiazolidin-3-yl)acetic acid derivatives (**A-1**–**A-3**) and photographs of the obtained compounds.

Derivatives of *(Z)-*2-(5-benzylidene-4-oxo-2-thioxothiazolidin-3-yl)acetic acid (**A-1**–**A-3**) were obtained by Knoevenagel condensation involving the reaction of the appropriate aldehyde (4-pyrrolidinobenzaldehyde—**A-1**, 4-(1H-pyrrol-1-yl)benzaldehyde—**A-2** or 4-(1H-imidazol-1-yl)benzaldehyde—**A-3**) with rhodanine-3-acetic acid, in the presence of ammonium acetate (Figure 1). Acetic acid was used as the solvent. The reaction mixtures were heated at reflux during the syntheses for four hours. The resulting sediments of raw products were filtered off and washed thoroughly with distilled water. This made removing the remains of ammonium acetate and acetic acid possible. Crystallization in acetonitrile was used to purify the obtained compounds from unreacted substrates. Due to the laboratory procedure used, the planned derivatives were synthesized with yields ranging from 54 to 71%. The structure of compounds **A-1**–**A-3** was confirmed by NMR spectroscopy (^1^H, ^1^H-^1^H COSY, ^13^C, ^1^H-^13^C HMQC and ^1^H-^13^C HMBC—all data are shown in ESI).

Moreover, the thermal properties of the obtained compounds were also examined. For this purpose, melting temperatures (T_m_) were measured, and thermogravimetric analysis (TGA) was performed. To characterize the thermal stability of the presented derivatives precisely, parameters such as 5% (T_5_) and 10% (T_10_) mass loss and the maximum decomposition temperature (T_max_) were determined. The results obtained are presented in Table 1 and Figure S1. In the case of melting temperature (T_m_), only the **A-2** derivative (T_m_ = 262 °C) had it. Moreover, all compounds (**A-1**–**A-3**) were characterized by high thermal stability above 240 °C. Compound **A-2** was the most thermally stable (T_5_ = 269 °C). The influence of the additional nitrogen atom in the aromatic imidazole ring is also clearly visible, which most likely reduces the thermal stability of the **A-3** derivative. This compound shows a 5% weight loss, approximately 20 °C lower than other derivatives. Interestingly, when analyzing the maximum distribution values, it can be seen that **A-3** has the best parameters (T_max_ = 307 °C). In turn, **A-1** completely decomposes at the lowest temperature of 284 °C.

### 3.2. DFT Calculations

#### 3.2.1. Chemical Properties

The charge-transfer (CT) excitation corresponds to the HOMO → LUMO transition (Figure S2). HOMO electrons are located on the benzylidene-4-oxo-2-thioxothiazolidin part, omitting the nitrogen atom and on the pyrrolidine (**A-1**), pyrrole (**A-2**) and imidazole (**A-3**) substituent rings. For **A-2** and **A-3**, the electron cloud extends over the entire ring, while for **A-1**, it is only on the nitrogen atom and neighboring carbon atoms. However, LUMO electrons move towards the benzylidene-4-oxo-2-thioxothiazolidin part, also covering the nitrogen atom. The electron cloud does not accumulate on the -COOH group, even though it belongs to moderately deactivating groups. For all derivatives, the energy separation between HOMO-LUMO orbitals (ΔE*_GAP_*, Table S1) decreases as a function of solvent polarity. The **A-3** derivative has the highest values and **A-1** has the lowest values. The ${∆∆E}_{GAP}$ difference between them is 0.44 eV in toluene and increases to 0.56 eV in DMSO.

Chemical hardness (*η*) and softness (*σ*) are indicators of a substance’s stability and chemical activity. The harder molecules are more stable but less chemically active, while the less hard molecules facilitate charge transfer and promote reactions. Greater softness corresponds to the enhanced activity of the system. Based on Table S1, *η* values, similarly to ΔE*_GAP_*, are the highest for **A-3** and decrease with increasing solvent polarity. *σ* values show the opposite trend. This indicates its superior activity compared to the other molecules. A soft molecule undergoes a uni-molecular reaction more readily than a hard molecule. The reactivity of a soft molecule is greater than that of a hard molecule if there is the necessity of transferring electrons for the reaction. A hard molecule resists changes in its electron number and electronic charge distribution. On the basis of the estimated hardness of the tested derivatives, we predict that there is minor change in the value of chemical hardness while changing the substituent attached to the benzene ring. The chemical potentials (*μ*) of the studied molecules are negative, indicating that the adsorption process is spontaneous. Higher *μ* values for **A-3** imply the absconding nature of an electron from an equilibrium system. The global electrophilicity (*ω*) values indicate that **A-3** is the most electrophilic in nature among the studied systems. A high electronegativity value suggests an easy formation of coordination bonds during various chemical processes. 

To predict reactive sites for electrophilic (negative/yellow and red zones) attack and nucleophilic (blue/positive zones) attack of the tested derivatives, the Molecular Electrostatic Potential (MEP) surfaces were calculated (Figure S3). The most negative zone is located on the oxygen atom of the -COOH group and attached to the thiazolidine ring by a double bond. The maximum positive site accumulates on the hydrogen atom of the -COOH group. Changing the substituents, which are also susceptible to nucleophilic attack, does not change the arrangement of these zones. For **A-1**, the zone for nucleophilic attack, with a smaller positive charge, also extends over the pyrrolidine ring. However, the presence of an additional nitrogen atom in the aromatic substituent makes this area susceptible to attack by the electrophile. In turn, replacement with an imidazole ring causes the nitrogen of this substituent to become the place with the largest concentration of negative charge.

The designated difference in total density computed for the ground and excited states (Δ*ρ*(r), Figure S4) suggests that the locations of the depletion sites (blue) and the density increment zones (purple) depend on the type of substituent attached to the benzene. Increment zones accumulate mainly on the benzylidene-4-oxo-2-thioxothiazolidin part and, to a small extent, on the -COOH group and substituents. In turn, the depletion sites for **A1** include the benzylidene-4-oxo-2-thioxothiazolidin part and the pyrrolidine ring, where they are significantly shifted towards the nitrogen atom and the carbon atoms are connected to it by a single bond. In the case of **A2**, the increment positions remain virtually unchanged. The differences are noticeable in the depletion zones, especially in the pyrrole ring. They appear on the nitrogen atom and two opposite carbon atoms (=C-C=). For the **A3** derivative, both zones disappear at the imidazole ring. Furthermore, the depletion zones disappear on benzene and are completely shifted to the thiazolidine ring, especially to the sulfur atom (=S). Table S2 shows the *D_CT_* values as a measure of the length of the electron transfer associated to an electronic transition. The index, based only on the computed electronic density for the ground and excited states, quantifies the charge-transfer (CT) length as the distance between the barycenters of the density increment and depletion regions upon electronic excitation. This index shows a monotonous increase with increasing polarity of the medium, and ${∆D}_{CT}^{DMSO-toluene}$ does not exceed 0.53 Å, 0.59 Å and 0.46 Å for **A-1**, **A-2** and **A-3**, respectively. The **A-2** derivative is described by the largest *D_CT_* values and **A-3** by the smallest ones. The ${∆D}_{CT}^{A2-A3}$ difference between them in toluene is 1.06 Å, increasing to 1.16 Å in DMSO. The *D_CT_* index confirms the CT character of the discussed derivatives and the contributions from the HOMO → LUMO transition. The amount of transferred charge increases monotonously as a function of environmental polarity for **A-1** and **A-2** and decreases for **A-3**. For **A-1** and **A-3** molecules, the ${∆q}_{CT}$ differences between extremely polar solvents do not exceed 0.006 e and 0.024 e for **A-2**.

The tested molecules are characterized by good solubility in all media, which is indicated by the free energy of solvation value (Δ*G_solv_*, Table S3). The presence of the imidazole ring maximizes the solubility, while the presence of the pyrrole ring minimizes it. The differences ${∆∆G}_{solv}^{A3-A2}$ are insignificant and amount to 3.54 kcal/mol and 3.71 kcal/mol in toluene and DMSO, respectively. For all derivatives, the Δ*G_solv_* values show non-monotone behavior as a function of the medium polarity.

**Figure 2:**
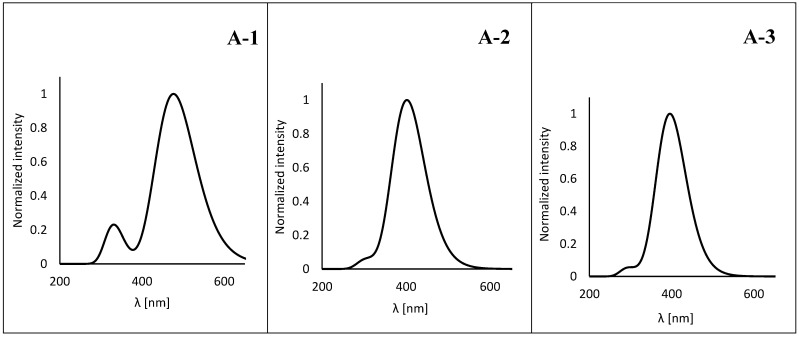
The vertical excitation energies designated theoretically.

The theoretical absorption maxima ($\lambda_{ABS}$) are shown in Table S4. According to the results obtained earlier [55,56,57,58,59], PBE0 gives the best agreement with the experimental values and should be considered as a reference. Comparing experimental measurements with vertical values, for **A-1** the PBE0 functional shifts the position of the maximum $\lambda_{ABS}$ towards longer waves, while for **A-2** and **A-3** it shifts towards longer waves. The relative error for the first derivative is 6.10 nm, while for the remaining ones it is 7.94 nm and 6.96 nm. The remaining functionals used significantly overestimate the excitation energy values. CAM-B3LYP and LC-ωPBE shift the maximum of the absorption bands bathochromically, with an error of 31.61 nm and 55.08 nm. In turn, B3LYP and HSEH1PBE show trends analogous to PBE0; however, the error value increases significantly to the level of 36.88 nm and 40.29 nm. For each molecule, $\lambda_{ABS}$ values, similar to experimental values, show non-monotonic behavior as a function of solvent polarity. More importantly, as predicted by Δ*ρ*(r), an additional low-intensity absorption band appears at 313.60 nm, 306.20 nm and 303.80 nm for **A-1**, **A-2** and **A-3**, respectively (Figure 2). This is due to additional contributions from other orbitals, HOMO-1 → LUMO for **A-1** (4%) and HOMO-2 → LUMO for **A-2** (3%) and **A-3** (4%). The excitation energy values determined using the cLR approximation ($\lambda_{cLR}$) are much more overestimated, compared to those measured experimentally, than those obtained using the TD-DFT method (Table S5). Maximum $\lambda_{cLR}$ values are bathochromically shifted relative to $\lambda_{ABS}$; however, a hypsochromic effect is observed for A1 in toluene and CHCl_3_. Also, the average ${\Delta \lambda }^{cLR-exp}$ error is higher and is 8.17 nm, 16.78 nm and 13.92 nm for **A-1**, **A-2** and **A-3**, respectively.

The determined values of dipole moments are not consistent with the behavior of the maxima of the absorption bands (Table S6). They increase monotonously with the increase in the polarity of the medium, which is characteristic for positive solvatochromism. The highest $\mu_{GS}$ and $\mu_{CT}$ values are characterized by **A-1**, and the lowest by **A-3**. The differences in toluene between them, ${∆\mu }_{GS}^{A1-A3}$ and ${∆\mu }_{CT}^{A1-A3}$, are 11.19 D and 16.21 D, increasing in DMSO to 13.16 D and 19.61 D. The **A-1** derivative is also described by the highest polarity of the CT state (${∆\mu }_{CT-GS}$). The presence of the pyrrole ring reduces the ${∆\mu }_{CT-GS}^{A1-A2}$ slightly; for toluene, the ${∆\mu }_{CT-GS}$ is 4.69 D and in DMSO it is only 0.09 D. In turn, for **A-3**, ${∆\mu }_{CT-GS}$ does not exceed the value of 2.30 D (in toluene) and shows non-monotonic behavior as a function of the environmental polarity.

Theoretically calculated NLO values are shown in Table S7. The values of $\langle \alpha \rangle$ and $\beta_{vec}$ increase monotonously as a function of the solvent polarity. Both $\langle \alpha \rangle$ and $\beta_{vec}$ values will increase monotonously as the polarity of the medium increases. Derivative **A-1** is characterized by the highest non-linear response, while **A-3** is characterized by the lowest. The $\langle \alpha \rangle$ values for **A-2** are lower than **A-1** by 5.74% in toluene and 8.91% in DMSO. For **A-3**, these differences are 10.60% and 14.14%. Changing the aromatic substituent has a greater impact on $\beta_{vec}$ values. Replacing the pyrrolidine ring to pyrrole reduces $\beta_{vec}$ by 28.13% in toluene and 41.40% in DMSO. In turn, replacement with an imidazole ring increases these differences to 61.71% and 72.31%, respectively. This indicates that the presence of the imidazole ring not only shifts the maximum of the absorption bands towards shorter wavelengths but also significantly reduces the values of dipole moments and suppresses the non-linear response of the system.

#### 3.2.2. Biological Properties

**Figure 3:**
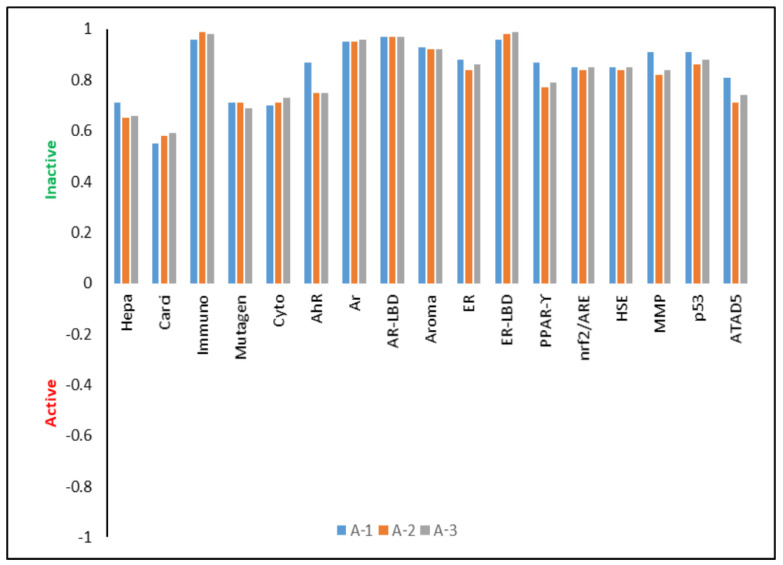
Biological activities. (Hepa-hepatotoxicity, Carci-carcinogenicity, Immuno-immunotoxicity, Mutagen-mutagenicity, Cyto-cytotoxicity, AhR-aryl hydrocarbon receptor, Ar-androgen receptor, AR-LBD-androgen receptor ligand-binding domain, Aroma-aromatase, ER-estrogen receptor alpha, ER-LBD-estrogen receptor ligand-binding domain, PPAR-γ-peroxisome proliferator-activated receptor gamma, nrf2/ARE-nuclear factor (erythroid-derived 2)-like 2/antioxidant responsive element, HSE-heat shock factor response element, MMP-heat shock factor response element, p53-phosphoprotein (tumor suppressor), ATAD5-ATPase family AAA domain-containing protein 5).

The tested derivatives are characterized by relatively good bioavailability. The theoretically calculated LogP value is 2.58, 2.70 and 2.35 (±0.25) for **A-1**, **A-2** and **A-3**, respectively. According to Lipiński’s rule [60,61], in each case it is less than 5, which confirms the ability of the tested compounds to cross cell membranes and the ability to bind to enzymes or receptors at the site of their action. The LogBCF values of −0.116, −0.018 and −0.047 indicate that the tested derivatives will not be bioaccumulative in living organisms and will be easily excreted in the urine. Described molecules are characterized by high metabolism by CYP450-2D6 (probability (P) is $P^{A_{1}}=$ 94.21%, $P^{A_{2}}=$ 93.78% and $P^{A_{3}}=$ 93.16%) and by CYP450-3A4 ($P^{A_{1}}=$ 92.45%, $P^{A_{2}}=$ 90.69% and $P^{A_{3}}=$ 91.81%). These quantities clearly indicate the rapid removal of compounds from tissues and the human body without interacting with other biomolecules and drugs. The theoretically determined oral toxicity LD_50_ values are 1345.00 mg/kg, 1929.00 mg/kg and 2180.00 mg/kg for **A-1**, **A-2** and **A-3**, respectively. These values indicate that the tested systems should be treated as practically non-toxic. For the intraperitoneal route of administration, LD_50_ values are 430.00 mg/kg, 562.00 mg/kg and 621.00 mg/kg; for the intravenous route of administration, LD_50_ values are 331.10 mg/kg, 410.15 mg/kg and 499.00 mg/kg; for the subcutaneous route of administration, LD_50_ values are 356.60 mg/kg, 541.00 and 637.50 mg/kg. Moreover, the tested compounds do not show mutagenic (the probability of occurrence is in the range of 69–71%), cytotoxic (70–73%), immunotoxic (96–99%), hepatoxic (65–71%) or carcinogenic (55–59%) properties (Figure 3). The tested derivatives also show many other biological properties related to nuclear receptor signaling pathways and stress response pathways, and changing the substituent does not significantly affect the probability of occurrence of a given activity. 

**Figure 4:**
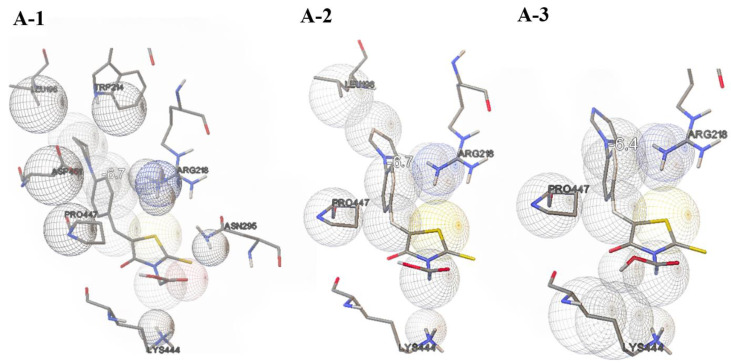
Molecular docking results.

Molecular docking results showed that the zone with the highest affinity for HSA is the active site at LYS444. For **A-3**, the affinity value (ΔG_b_) for the protein is −6.4 kcal/mol with an inhibition constant (*K*_i_) of 0.65 mM. For the other two derivatives, ΔG_b_ is −6.7 kcal/mol and *K*_i_ is 0.58 mM and 0.61 mM for **A-1** and **A-2**, respectively. Molecules align with the -COOH group towards the amino group of the protein (Figure 4). This orientation is justified because, according to the description presented by Brinkley [62], a bioconjugate is created by forming a bond between the carbon atom of the -COOH group and the nitrogen atom of the amino group. In each case, the biocomplex is not stabilized by the formation of *H*-bonds and *π–π* interactions. For each derivative, apart from LYS444, the aromatic cavity is also formed by PRO477 and ARG218. In the case of **A-1** and **A-2**, there is also an interaction between LEU198 and the aromatic substituent. This suggests that the presence of a nitrogen atom in the imidazole ring blocks this interaction. Moreover, for **A-1**, TRP214 and ASP451 are also observed to be close to the pyrrolidine ring, which is related to the lack of double bonds in the aromatic ring. After spatial adjustment to the active site, the tested compounds undergo structural changes (Figure S5). In each case, the -CH_2_-COOH group rotates by 180° on the C-N(thiazole) bond relative to the plane of the molecule. Another rotation takes place on the C(thiazole)=C bond, causing the benzene-aromatic substituent part to rotate, from 76.23° for **A-1** to 78.54° for **A-3**. Breaking the planarity in this way does not affect the dihedral angle S-C(thiazole)=C-C(benzene), and the differences $\Delta^{o}$ between the initial structure and that after molecular docking are 0.067°, 0.039° and 0.006° for **A-1**, **A-2** and **A-3**, respectively. Similar rotations of molecules when fitting into the aromatic cavity when forming active marker–protein complexes have been observed for other classes of compounds, such as luciferin derivatives [63]. A C=C double bond is not rigid and inflexible. Significant distortions of a double bond are possible without fatal reduction in the strength of the π bond. This is due to the pyramidalization of sp^2^-hybridized carbon atoms that accompanies twisting [64]. This ensures optimal alignment of the fluorescent probe with the active site. The spatial alignment of the molecules to the aromatic center also results in the rotation of the aromatic substituent on the C(benzene)-N bond. In this case, the rotation is also accompanied by a change in the C-C(benzene)-N-C(substituent) dihedral angle of 32.970°, 31.282° and 17.922° for **A-1**, **A-2** and **A-3**, respectively. In the last case, the smallest $\Delta^{o}$ value results from the previously described smallest number of interactions with HSA amino acids. In our case, the use of molecular docking with the use of AutoDock Vina is to indicate the regions and specificity of the potential interaction in the probe–protein system and are illustrative. Detailed research for a larger group of this class of compounds using molecular dynamics will constitute a separate scientific publication.

### 3.3. Optical Properties

**Figure 5:**
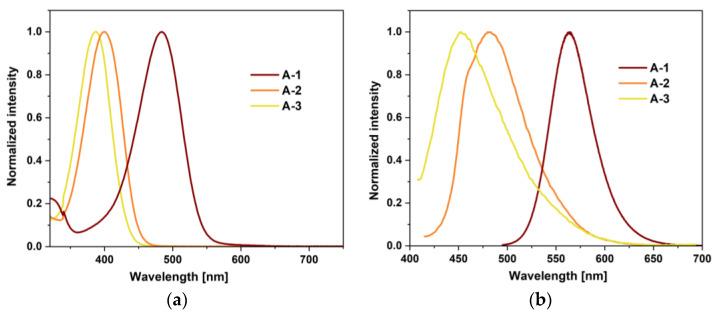
(**a**) Absorption and (**b**) emission spectra of **A-1**–**A-3** recorded in DMSO.

To ensure better characterization of compounds (**A-1**−**A-3**), the tests were performed in six organic solvents. The concentration of the samples was 2.5 × 10^−5^ mol/L. In addition, a solubility test of the sample compounds in water was performed. The collected data are presented in Table 2. All derivatives were insoluble in water. As a consequence, research in this environment was not possible. Moreover, **A-3** was insoluble in toluene and acetonitrile. An absorption peak was recorded in solvents in which the samples were soluble. For compounds **A-2** and **A-3**, it was in the range of 369–400 nm. For compound **A-1**, this peak was bathochromically shifted and was in the 460–482 nm range (Figure 5a). Compound **A-1** exhibits a solvatochromic effect in UV–Vis spectra. As the solvent’s polarity increases, the band’s redshift is noticeable. This is typical of the ICT transition with an increase in the dipolar moment upon excitation [65,66], which results in more excellent stabilization of the more polar excited state in polar solvents [67]. For molecules **A-2** and **A-3**, the solvatochromic effect was much less noticeable. The absorption of these compounds also results from intramolecular charge transfer (ICT) between the substituent and rhodanine-3-acetic acid via the π bridge [7]. Analyzing the location of the absorption bands of all derivatives, we see that the highest redshift characterizes **A-1** (Figure 5a). This is due to the strong ability of the pyrrolidine unit to donate electrons to the acceptor via the benzene π bridge. In the case of **A-2** and **A-3**, there is a significant hypsochromic shift relative to **A-1**. This may be due to the more planar nature of the pyrrole and imidazole rings. Such planarity may promote the delocalization of the lone electron pair of the nitrogen atom, which leads to a reduction in the electron-pushing effect [7].

Then, emission maxima were determined by continuing measurements in solvents of different polarities (Figure 5b and Table S8). The measured molecules showed weak fluorescence intensity, which is consistent with the literature for structurally similar compounds [7]. In the case of **A-1**, literature reports indicate that it has a quantum efficiency of 0.0019 and a small decay time in methanol [7]. Moreover, quantum yields in the DMSO solution were determined for all derivatives in accordance with the literature [27]. These yields were relatively small. **A-1** had the highest efficiency of 0.078. Then, a decrease in performance was observed in the order **A-1** (0.078) > **A-2** (0.025) > **A-3** (0.016). As the emission bands shift towards blue, we observe a decrease in the photoluminescence quantum yield. The weak emission of all compounds, the determined quantum yields and the literature parameters of **A-1** suggest that the emission bands can also be attributed to intramolecular charge transfer (ICT). As is known, ICT is a competitive relaxation process of the singlet excited state, which may result in a decrease in fluorescence [68]. For the series of compounds **A-1**–**A-3**, changes in the position of the peak maxima could also be observed depending on the solvent used. As the polarity of the solvent increases, a red shift is observed. The highest solvatochromic effect was observed for compound **A-1** (Table S8). Moreover, this effect is also a result of ICT because compounds of this nature are sensitive to changes in solvent polarity. For all derivatives, the Stokes shift was determined. Analyzing this parameter, we see large shifts resulting from the effective transfer of charge from the substituent (donor) to rhodanine-3-acetic acid (acceptor). To better represent the solvatochromic effect, an analysis using the Lippert–Mataga equation was performed to estimate the difference between the excited dipole moments and the ground states (Δ*μ* = *μ_e_* – *μ_g_*) by plotting the Stokes shifts Δ*E_exc-em_* (cm^−1^) against the orientation polarization (Δ*f*) solvents. The relation between the Stokes shifts and solvent polarizability can be expressed by the following equation:

$$∆E_{exc-em}=\frac{2{\left(\mu_{e}-\mu_{g}\right)}^{2}}{hca^{3}}∆f+Const.$$

To understand the equation used, you need to explain its elements. So, “*h*” is Planck’s constant, “*c*” is the speed of light in a vacuum, and “*a*” stands for the Onsager cavity radii. The value of “*a*” was calculated theoretically and is 5.60 Å for **A-1** and 5.53 Å for **A-2**. The orientation polarizability (∆f) of the solvent was calculated by the following equation:

$$∆f=\frac{\varepsilon -1}{2\varepsilon +1}\;–\;\frac{n^{2}-1}{2n^{2}+1}$$

where *ε* is the dielectric constant of the solvent and *n* is the optical refractive index of the solvent. Due to the low solubility and low emissivity of compound **A-3**, it was impossible to determine its dipole moment. The calculated values of the dipole moments for **A-1** and **A-2** were 8.83 D and 8.47 D, respectively. A graphical representation of the Lippert–Mataga plots is provided in the Supplementary Materials (Table S10).

Next, the stability of the solutions over time was tested. Measurements were performed in DMSO solution at room temperature. For all molecules, the absorption profile did not change over time. This proves the stability of the tested compounds in the solution. The obtained results are presented in Table S9.

### 3.4. Bioimaging

#### 3.4.1. Cytotoxicity 

**Figure 6:**
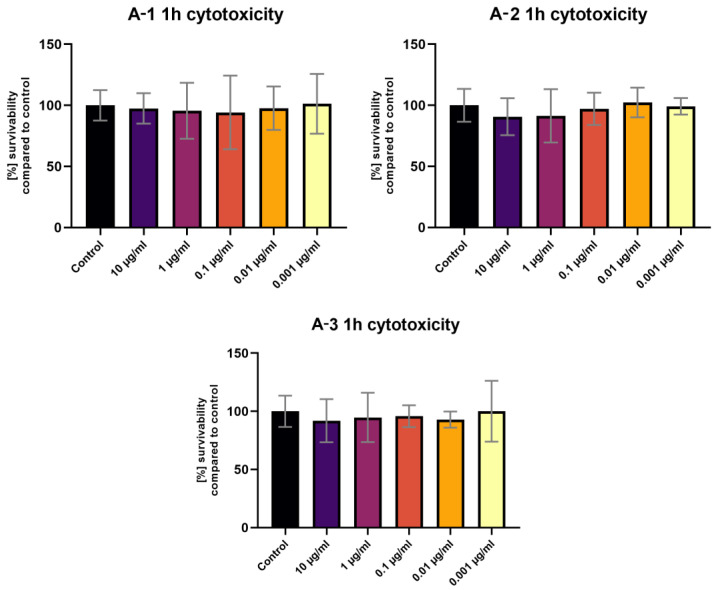
The 3T3 MTT assay results after 1 h of exposure to various **A-1**–**A-3** concentrations. Results presented as % of survivability of untreated control. Statistical test used—one-way ANOVA with Dunnett’s post hoc (Cl at 95%).

**Figure 7:**
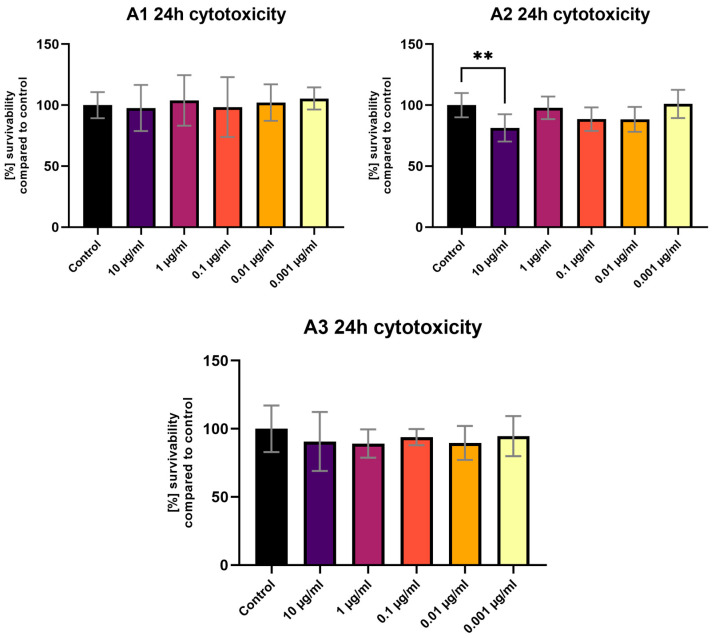
The 3T3 MTT assay results after 24 h of exposure to various **A-1**–**A-3** concentrations. Results presented as % of survivability of untreated control. Results, significantly different from that of the control, are marked with [**]. Statistical test used—one-way ANOVA with Dunnett’s post hoc (Cl at 95%).

The MTT assay performed after a 1 h exposure of the tested compounds showed no statistically significant differences in viability compared to the control (Figure 6). The 24 h exposure (Figure 7) revealed a statistically significant decrease in viability for the highest tested concentration (10 μg/mL) of the **A-2** compound (Figure 6). No fluorescence of cells in the live culture was observed after 1 h and 24 h of staining using tested compounds. Background fluorescence levels were high for **A-1** and **A-2**.

#### 3.4.2. Imaging 

**Figure 8:**
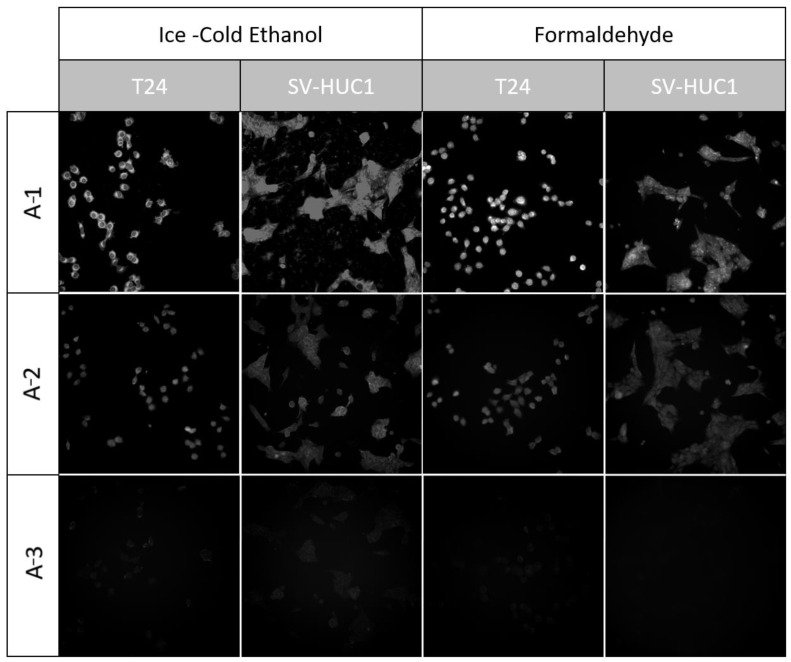
Fluorescence images acquired using the U-FBNA filter set and 450 nm LED—the equivalent of a narrow-band FITC channel. Images taken from T24 and SV-HUC1 cell lines were stained with the tested compounds after fixation with ethanol and formaldehyde. All images were acquired using an IX83 microscope (Olympus, Tokyo, Japan) and LT3 plus monochromatic camera (Hamamatsu, Shizuoka, Japan).

Both of the assessed cell fixation methods, 96% ice-cold ethanol and 4% formaldehyde, were compatible with the **A-1** and **A-2** compounds. During observation, cells stained with the **A-3** compound showed only minor fluorescence using both fixation methods. The formaldehyde fixation enabled more uniform staining and preservation of cell morphology. No significant differences in staining effectiveness were noted between T24 and SV-HUC1 cell lines (Figure 8). After staining, none of the tested compounds presented fluorescence in the UV or 647/Cy3 channels.

#### 3.4.3. Compound Compatibility with Nuclear Stains and Mounting Methods

**Figure 9:**
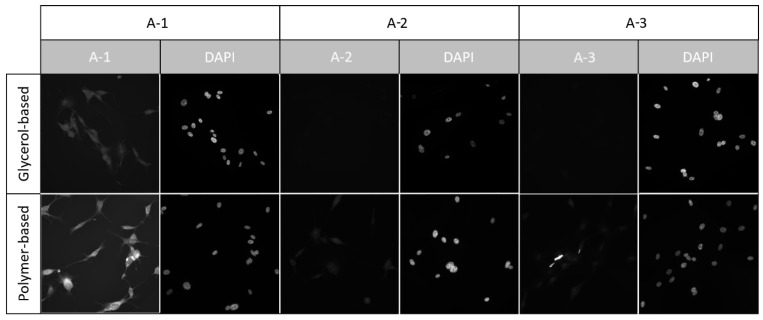
Fluorescence images were acquired using the U-FBNA filter set and 450 nm LED, equivalent to a narrow-band FITC channel, and U-FUNA narrow-band UV filter set with 365 nm LED excitation. Images were taken from the 3T3 cell line cultured on 0.17 mm imaging coverslips and stained with the tested compounds after fixation with formaldehyde and mounted using a glycerol-based mounting medium (Vectashield Vibrance, Vector) and polymer-based mounting medium (Eukitt, Sigma, Tokyo, Japan). All images were acquired using an IX83 microscope (Olympus, Japan) and LT3 plus monochromatic camera (Hamamatsu).

**Figure 10:**
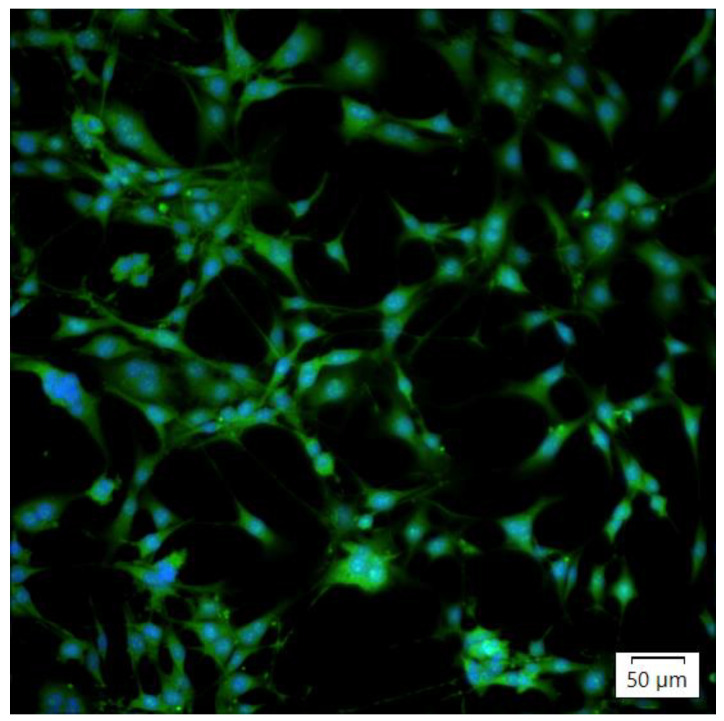
Image of 3T3 cells stained with A1 and DAPI after formaldehyde fixation. The image consists of two merged fluorescence channels, and a pseudo color mask was added to visualize the actual fluorescence colors visible. The image was acquired using an IX83 microscope (Olympus, Japan) and LT3 plus monochromatic camera (Hamamatsu, Shizuoka, Japan).

None of the tested compounds interfered with imaging via the UV channel. The nuclear stain (DAPI) was visible, and no artifacts were present (Figure 9 and Figure 10). The **A-1** compound was compatible with both mounting methods, while the **A-2** and **A-3** stains suffered a decrease in fluorescence intensity, especially with the glycerol-based mounting medium. We have also observed that using a glycerol-based mounting medium caused a positive correlation between the colocalization of the DAPI stain and the tested compounds with increased fluorescence in the FITC channel in the nucleus area.

### 3.5. Antimicrobial Effect

The antimicrobial activity of the newly synthesized compounds **A-1**–**A-3** was tested towards reference Gram-positive (eight strains) and Gram-negative (five strains) bacteria. The antifungal effect against yeasts belonging to *Candida* spp. (five strains) was also investigated.

As presented in Table 3 and Table 4, these compounds showed some antimicrobial effects at MIC in the range 62.5–>2000 µg/mL. The reference strains of Gram-positive bacteria were more sensitive to the studied compounds (MIC = 62.5–1000 µg/mL, MBC = 125–>2000 µg/mL) than Gram-negative microorganisms (MIC = 500–>2000 µg/mL, MBC ≥ 2000 µg/mL). Among these substances, **A-1** and **A-2** indicated the highest antibacterial activity with MIC = 62.5–500 µg/mL. Its activity towards reference bacteria belonging to *Staphylococcus* spp., *Enterococcus faecalis*, *Micrococcus luteus* and *Bacillus* spp. was good or moderate. *M. luteus* ATCC 10240 was the most susceptible to these substances at MIC = 62.5 µg/mL. Moreover, the MBC values of compounds were in the range 125–>1000 µg/mL and MBC/MIC = 1–>16. These ratios show its bactericidal or bacteriostatic effect (Table 3).

The case of Gram-negative bacteria was similar. Only compounds **A-1** and **A-2** indicated mild or moderate bioactivity, with MIC = 500–1000 µg/mL. The substance **A-3** was inactive (MIC ≥ 2000 µg/mL) against these microorganisms. For all Gram-negative bacteria, MBC > 2000 µg/mL and MBC/MIC = >1, >2 or >4, respectively (Table 3).

The sensitivity of the fungi, belonging to reference strains from *Candida* spp., to the tested substances was similar. The compounds showed mild activity (MIC = 1000 µg/mL) or no bioactivity (MIC = 2000 µg/mL) towards these yeasts. Their MFC values were the same or 2-fold higher than the MIC (MFC = 1000–2000 µg/mL), indicating an MFC/MIC index = 1–2 and its fungicidal effect (Table 4).

## 4. Conclusions

To sum up, as a result of Knoevenagel condensations, three derivatives of 2-(5-benzylidene-4-oxo-2-thioxothiazolidin-3-yl)acetic acid (**A-1**–**A-3**) were obtained, with good yields in the range of 54–71%. Moreover, all synthesized compounds showed thermal stability above 240 °C. All compounds were characterized by one absorption and emission peak. This peak could be attributed to intramolecular charge transfer (ICT). Compound **A-1** exhibited the most bathochromic shift. Moreover, this molecule exhibited solvatochromic properties. The **A-1**–**A-3** derivatives showed stability in the DMSO solution about measurements performed over 24 h. The **A-1** and **A-2** compounds were successfully used as fluorescent stains for fixed cells of mammalian origin. After cell staining, those two compounds presented fluorescence in the FITC and TRITC channels, but no fluorescence was detected in the UV and 647/Cy3 channels. The tested compounds did not demonstrate effectiveness in live-cell staining and, therefore, are unsuitable as cell trackers even though they show no cytotoxic properties in the MTT assay. Since no fluorescence was present during live-cell staining and we observed fluorescence when using fixed cells, there is a possibility of using those compounds as potential cell survivability markers in routine live-dead assays. **A-1** and **A-2** might also be used as counter-stains during HCS image analysis since they stain the entire cell cytoplasm. Both **A-1** and **A-2** were compatible with formaldehyde and ethanol fixation methods, two of the most popular mounting methods used for microscope imaging. Formaldehyde fixation paired with a polymer-based mounting medium presented the best overall staining results and compatibility with UV nuclear stains. Our testing confirmed that **A-1** and **A-2** are compatible with cell staining performed on glass and polymer microplates. Our results indicated that among the newly synthesized compounds, **A-1** and **A-2** showed the highest antimicrobial effect, especially against Gram-positive bacteria. Their activity was good or moderate towards these bacteria with MIC in the range from 62.5 to 500 µg/mL. These results showed satisfactory activity and the potential antibacterial application of some newly synthesized compounds.

## Figures and Tables

**Table 1:** Thermal stability of **A-1**–**A-3**.

Code	T_m_ ^1^ [°C]	T_5_ ^2^ [°C]	T_10_ ^2^ [°C]	T_max_ ^3^ [°C]
**A-1**	-	261	276	284
**A-2**	262	269	278	287; 307
**A-3**	-	243	303	307

^1^ T_m_ is the melting point. ^2^ T_5_ and T_10_ are temperature at 5% and 10% weight loss, respectively. ^3^ The temperature of the maximum decomposition rate.

**Table 2:** Collected data on photophysical properties for **A-1**–**A-3**.

Compound	Solvent	Absorption ^1^ (λ_abs_) [nm]	Emission (λ_em_) [nm]	Stokes Shift ^2^ [nm]
**A-1**	Toluene	469	511	42
	THF	470	532	62
	CHCl_3_	479	537	58
	MeCN	476	557	81
	DMSO	482	563	81
	MeOH	460	550	90
	H_2_O	-	-	-
**A-2**	Toluene	400	463	63
	THF	393	468	75
	CHCl_3_	397	473	76
	MeCN	390	477	87
	DMSO	400	482	82
	MeOH	390	464	74
	H_2_O	-	-	-
**A-3**	Toluene	-	-	-
	THF	380	452	nd
	CHCl_3_	369	415	46
	MeCN	-	-	-
	DMSO	386	453	67
	MeOH	374	468	94
	H_2_O	-	-	-

^1^ The bold wavelengths were taken to register the emission spectrum, ^2^ λ_em_–λ_abs_.

**Table 3:** The activity data of the tested compounds **A-1**–**A-3** expressed as MIC and MBC [µg/mL] values and MBC/MIC ratios against the reference strains of bacteria. The standard antibiotics—ciprofloxacin (CIP) and vancomycin (VA *)—were used as positive controls.

Species		MIC and MBC [µg/mL] Values and MBC/MIC Ratios of the Studied Compounds and Positive Controls											
		A-1			A-2			A-3			CIP/VA *		
		MIC	MBC	MBC/MIC	MIC	MBC	MBC/MIC	MIC	MBC	MBC/MIC	MIC	MBC	MBC/MIC
**Gram-positive bacteria**	*Staphylococcus aureus* MRSA ATCC 43300	125	1000	8	250	>2000	>4	1000	>2000	>2	0.24	0.24	1
	*Staphylococcus aureus* MSSA ATCC 29213	250	2000	8	250	2000	8	1000	>2000	>2	0.48	0.48	1
	*Staphylococcus aureus* MSSA ATCC 25923	125	>2000	>16	500	2000	16	1000	>2000	>2	0.48	0.48	1
	*Staphylococcus epidermidis* ATCC 12228	250	>2000	>8	250	>2000	>8	1000	>2000	>2	0.12	0.12	1
	*Enterococcus faecalis* ATCC 29212	250	>2000	>8	125	>2000	>16	1000	>2000	>2	0.98 *	1.95 *	2 *
	*Micrococcus luteus* ATCC 10240	62.5	1000	16	62.5	2000	32	500	500	1	0.98	1.98	2
	*Bacillus subtilis* ATCC 6633	250	250	1	125	125	1	500	>2000	>4	0.03	0.03	1
	*Bacillus cereus* ATCC 10876	500	1000	2	250	2000	8	1000	2000	2	0.06	0.12	2
**Gram-negative bacteria**	*Escherichia coli* ATCC 25922	1000	>2000	>2	1000	>2000	>2	2000	>2000	>1	0.004	0.004	1
	*Klebsiella pneumoniae* ATCC 13883	1000	>2000	>2	1000	>2000	>2	2000	>2000	>1	0.12	0.12	1
	*Proteus mirabilis* ATCC 12453	1000	>2000	>2	1000	>2000	>2	2000	>2000	>1	0.03	0.03	1
	*Salmonella* Typhimurium ATCC 14028	500	>2000	>4	500	>2000	>4	>2000	>2000	>1	0.06	0.06	1
	*Pseudomonas aeruginosa* ATCC 27853	500	>2000	>4	1000	>2000	>2	2000	>2000	>1	0.48	0.98	2

**Table 4:** The activity data of the tested compounds **A-1**–**A-3** expressed as MIC and MFC [µg/mL] values and MFC/MIC ratios against the reference strains of fungi. The standard antibiotic—nystatin (NY)—was used as a positive control.

Species		MIC and MFC [µg/mL] Values and MFC/MIC Ratios of the Studied Compounds and Positive Control											
		A-1			A-2			A-3			NY		
		MIC	MFC	MFC/ MIC	MIC	MFC	MFC/ MIC	MIC	MFC	MFC/ MIC	MIC	MFC	MFC/ MIC
**Fungi**	*Candida albicans* ATCC 2091	1000	1000	1	1000	2000	2	1000	1000	1	0.24	0.24	1
	*Candida albicans* ATCC 10231	1000	1000	1	1000	2000	2	1000	1000	1	0.48	0.48	1
	*Candida parapsilosis* ATCC 2201	1000	2000	2	2000	2000	1	1000	2000	2	0.24	0.48	2
	*Candida glabrata* ATCC 90030	1000	2000	4	2000	2000	1	2000	2000	1	0.24	0.48	2
	*Candida krusei* ATCC 14243	1000	2000	4	2000	2000	1	2000	2000	1	0.24	0.24	1

## Data Availability

The data presented in this study are available in the Supplementary Material.

## Supplementary Materials

The following supporting information can be downloaded at: https://www.mdpi.com/article/10.3390/s24051524/s1, Measurements and general methods; Synthesis, NMR and HRMS spectra; Table S1: The frontier orbital energies in tested solvents for Z-isomers. Values are given in [eV]; Table S2: CT parameters for the bright low-lying excited state. qCT values are given in [e] and DCT in [Å]; Table S3: Free energies (ΔGsolv, kcal/mol) of solvation; Table S4: The vertical excitation energies (in nm); Table S5: The cLR-corrected excitation energies (in nm); Table S6: The calculated values of dipole moments (in D) for the ground and CT excited state; Table S7: The non-linear properties of tested isomers. Values are given in (a.u.); Table S8: Absorption and emission of **A-1**–**A-3**; Table S9: UV–Vis spectra in DMSO recorded at time intervals at room temperature; Table S10: The Lippert-Mataga plots; Figure S1: TGA—thermal properties of **A-1**–**A-3**; Figure S2: HOMO/LUMO plots; Figure S3: The MEP surfaces; Figure S4: Density difference plots; Figure S5: Structural changes of the tested derivatives after spatial adjustment to the protein.
