# Supplementary material for: Can a Small Change in the Heterocyclic Substituent Significantly Impact the Physicochemical and Biological Properties of (Z)-2-(5-Benzylidene-4-oxo-2-thioxothiazolidin-3-yl)acetic Acid Derivatives?
Source: Sensors (Basel). 2024 Feb 27;24(5):1524. doi: 10.3390/s24051524 (PMC10935081; doi:10.3390/s24051524)
Supplement: Supplementary file 1 [file sensors-24-01524-s001.zip › sensors-2826695-supplementary.pdf]

Supporting Information

Can a Small Change in the Heterocyclic Substituent Significantly Impact the Physicochemical and Biological Properties of (Z)-2-(5-Benzylidene-4-oxo-2-thioxothiazolidin-3-yl)acetic Acid Derivatives?

Agata Szlapa-Kula [1,*], Slawomir Kula [1,*], Łukasz Kaźmierski [2], Anna Biernasiuk [3] and Przemysław Krawczyk [4]

[1] Institute of Chemistry, Faculty of Science and Technology, University of Silesia in Katowice, Szkolna 9 St., 40-007 Katowice, Poland

[2] Urology and Andrology, Department of Tissue Engineering, Collegium Medicum, Nicolaus Copernicus University, M. Curie Skłodowskiej 9, 85-094 Bydgoszcz, Poland

[3] Department of Pharmaceutical Microbiology, Faculty of Pharmacy, Medical University of Lublin, 20-093 Lublin, Poland

[4] Department of Physical Chemistry, Faculty of Pharmacy, Collegium Medicum, Nicolaus Copernicus University, Kurpińskiego 5, 85-950 Bydgoszcz, Poland

* Correspondence: agata.szlapa-kula@us.edu.pl (A.S.-K.); slawomir.kula@us.edu.pl (S.K.)

Table of Contents

1. **Materials** 2
2. **Measurements and general methods** 2
3. **Synthesis, NMR and HRMS spectra** 5
4. **Thermal properties** 15
5. **DFT calculations** 15
6. **Optical properties** 21
7. **Literature** 23

1. Materials

4-Pyrrolidinobenzaldehyde (98%, Combi-Blocks), 4-(1H-Pyrrol-1-yl)benzaldehyde (98%, Combi-Blocks), 4-(1H-Imidazol-1-yl)benzaldehyde (98%, Combi-Blocks), rhodanine-3-acetic acid (>98.0%, TCI), ammonium acetate (puriss. p.a., ACS reagent, reag. Ph. Eur., ≥98%, Merck-Sigma-Aldrich), DMSO-d_6 (99.9 atom % D, Merck-Sigma-Aldrich), acetonitrile (pure for analysis, Chempur), acetonitrile (for HPLC-GC, ≥99.8% (GC), Merck-Sigma-Aldrich), dichloromethane (for HPLC, ≥99.8%, contains amylene as stabilizer, Merck-Sigma-Aldrich), chloroform (for HPLC, ≥99.8%, amylene stabilized, Merck-Sigma-Aldrich), methanol (for HPLC, ≥99.9%, Merck-Sigma-Aldrich), DMSO (pure for analysis, Chempur), THF (pure for analysis, Chempur), Toluene (pure for analysis, Chempur), glacial acetic acid (99,5% pure p. a, Chempur).

2. Measurements and general methods

Bruker Avance 400 MHz instrument used to record the NMR spectra in DMSO-d6 (as solvent). Thermogravimetric analysis was performed on Perkin Elmer Thermogravimetric Analyzer Pyris 1 TGA at a heating rate of 15°C/min under nitrogen. UV/Vis spectra were recorded with a Biosens model UV 5600 UV/Vis spectrophotometer. Photoluminescence emission spectra were acquired using Hitachi Fluorescence Spectrophotometer F-7100. The photoluminescence quantum yields of the *(Z)*-2-(5-benzylidene-4-oxo-2-thioxothiazolidin-3-yl)acetic acid derivatives were determined relative to Rhodamine B in methanol as a standard (Φ_{ref} =0.7)[1], using the literature procedure[1].

In vitro culture

Mouse fibroblasts (3T3 – NIH3) were used as a model cell line for compound cytotoxicity testing. T24 (Human, Urinary bladder Transitional Cell Carcinoma) and SV-HUC1 (Human, uroepithelium epithelial cell line) were used for testing staining procedures for fixed cells. Before the experiment started, the cell lines were in culture for at least five passages. Standard, recommended culture conditions were applied: 37°C, 5% CO_2, 98% humidity, DMEM/F12K (Corning USA) medium was used for 3T3 and T24 cells, for SV-HUC1 RPMI medium was the dedicated choice (Corning USA). All media were supplemented with 10% FBS (Corning USA) and a recommended dose of antibiotics.

Stock reagent preparation

The stock solution of all reagents was prepared in cell culture certified DMSO (Dimethyl sulfoxide, BioUltra purity, Merck, USA). After dissolving the compounds, they were centrifuged for 5 minutes at 1000xg and filtered using a 0,22 µm syringe filter (PES Millex-GP).

Cytotoxicity assay and Live-cell staining

To determine the cytotoxicity of the tested compounds, we evaluated both short-term (1h) and long-term (24h) expositions toxicity starting from the dilution of 10µg of substance per 1 ml of culture media. After the exposition phase, the MTT assay was performed for short-term and long-term experiments. Before exposition, cells were seeded in 96 microplates (Nunc FB TC) at a density of 8000 cells/well and pre-incubated for 24 hours before the addition of the tested compound. After the pre-incubation, the medium was discarded, and all wells were washed twice with PBS. Tested compounds were pre-diluted in culture medium at a range of concentrations (20µg/ml to 0,002 µg/ml). After the exposition period, the tested compounds were discarded, and all wells were washed 2x with PBS. Directly after that, the MTT assay procedure was performed.

To observe the live-cell staining capabilities of the tested compounds, we have conducted observations during the exposition phase of the MTT assay. Cell staining progress was monitored using a fluorescence microscope (IX83, Olympus) after 1h and 24h of staining.

The MTT assay starts with the addition of the MTT reagent (Thiazolyl Blue Tetrazolium Bromide, Sigma) at a concentration of 1mg/ml diluted in DMEM medium without phenol red. After 2 hours of incubation, the MTT solution is discarded, and 100µl of DMSO is added. The absorbance is read at 570nm using a multi-plate reader (Multiskan Sky, Thermo, USA). The results presented contain % survivability based on the acquired absorbance compared to the untreated control.

Imaging

Before performing the imaging step, we assessed the effect of the fixation method on the staining effectiveness. For that, we fixed cells with 4% Formaldehyde (methanol-free) and with ice-cold 96% ethanol for 15 minutes. After two PBS washes, we left the cells for 24 hours with a 0.1% TRITON-X solution. After this incubation, the cells were again washed with PBS, and staining solutions were added. The staining solutions contained 1µg of the tested compounds per 1 ml PBS.

Fluorescence imaging of all samples was performed using a fully motorized, inverted IX83 microscope (Olympus, Japan) with Orca Flash LT3 Plus monochromatic camera

(Hamamatsu, Japan). An LED light source - pE-300 ultra (Colled, Great Britain)- was used to ensure low photobleaching and narrow excitation wavelengths. U-FUNA, U-FBNA, U-FGW, and U-FMCHE filter cube custom-made equivalents were used (Olympus, Japan) for the channels correlating to the UV, FITC, TRITC, and 647/Cy3 channels. Focusing was performed with a semi-automated system using laser autofocus (Z-Drift Compensation Unit, Olympus, Japan).

Compound compatibility with nuclear stains and mounting methods

Cells were prepared the same way as in the Imaging Paragraph, with the difference being the growth surface. Cells were cultured on imaging coverslip glasses. After fixation with 4% Formaldehyde, 10µg/ml of the tested compounds were used with the DAPI stain (300nM) for 60 minutes, and cells were washed with PBS afterward. Next, the coverslips were mounted with a non-aqueous (Eutkitt) and aqueous (Vectashield Vibrance) mounting media to assess their compatibility.

In vitro antimicrobial assay

The examined compounds **A-1** – **A-3** were screened *in vitro* for antibacterial and antifungal activities using the broth microdilution method according to European Committee on Antimicrobial Susceptibility Testing (EUCAST) [2] and Clinical and Laboratory Standards Institute (CLSI) guidelines [3] against a panel of reference strains of microorganisms, including Gram-positive bacteria (*Staphylococcus aureus* ATCC 43300 (Methicillin Resistant *S. aureus* – MRSA), *Staphylococcus aureus* ATCC 29213 (Methicillin Susceptible *S. aureus* – MSSA), *Staphylococcus aureus* ATCC 25923 (Methicillin Susceptible *S. aureus* – MSSA), *Staphylococcus epidermidis* ATCC 12228, *Enterococcus faecalis* ATCC 29212, *Micrococcus luteus* ATCC 10240, *Bacillus subtilis* ATCC 6633 and *Bacillus cereus* ATCC 10876), Gram-negative bacteria (*Escherichia coli* ATCC 25922, *Klebsiella pneumoniae* ATCC 13883, *Proteus mirabilis* ATCC 12453, *Salmonella* Typhimurium ATCC 14028 and *Pseudomonas aeruginosa* ATCC 9027) and fungi belonging to yeasts (*Candida albicans* ATCC 2091, *Candida albicans* ATCC 10231, *Candida parapsilosis* ATCC 22019, *Candida glabrata* ATCC 90030 and *Candida krusei* ATCC 14243). The microorganisms came from American Type Culture Collection (ATCC), routinely used for the evaluation of antimicrobials. All the used microbial cultures were first subcultured on nutrient agar or Sabouraud agar at 35°C for 18-24 h or 30°C for 24-48 h for bacteria and fungi, respectively.

Samples containing examined compounds **A-1** – **A-3** were first dissolved in dimethyl sulfoxide – DMSO (20 mg/mL). Subsequently MIC (Minimal Inhibitory Concentration) of

these substances was examined by the microdilution broth method, using their two-fold dilutions in Mueller-Hinton broth (for bacteria) and RPMI 1640 broth with MOPS (for fungi) prepared in 96-well polystyrene plates. Final concentrations of the compounds ranged from 2000 to 15.62 µg/mL. Microbial suspensions were prepared in 0.85% NaCl with an optical density of 0.5 McFarland standard. Next bacterial or fungal suspensions were added per each well containing broth and various concentrations of the examined compounds. After incubation (under the same conditions as before), the MIC was assessed spectrophotometric as the lowest concentration of the samples showing complete bacterial or fungal growth inhibition. Appropriate, growth and sterile controls were carried out. The medium with no tested substances was used also as control. The inhibition of microbial growth was judged by comparison with a control culture prepared without any sample tested. Ciprofloxacin, vancomycin or nystatin (Sigma-Aldrich Chemicals, St. Louis, MO, USA) were used as a reference antibacterial or antifungal compounds, respectively.

The MBC (Minimal Bactericidal Concentration) or MFC (Minimal Fungicidal Concentration) are defined as the lowest concentration of the compounds that is required to kill a particular bacterial or fungal species. MBC or MFC was determined by removing the culture using for MIC determinations from each well and spotting onto appropriate agar medium. Next, the plates were incubated. The lowest compounds concentrations with no visible growth observed were assessed as a bactericidal or fungicidal concentrations. All the experiments were repeated three times and representative data are presented [4,5].

In this study, no bioactivity was defined as a MIC > 1000 µg/mL, mild bioactivity as a MIC in the range 501 – 1000 µg/mL, moderate bioactivity with MIC from 126 to 500 µg/mL, good bioactivity as a MIC in the range 26 – 125 µg/mL, strong bioactivity with MIC between 10 and 25 µg/mL and very strong bioactivity as a MIC < 10 µg/mL [6]. The MBC/MIC or MFC/MIC ratios were calculated in order to determine bactericidal/fungicidal (MBC/MIC ≤ 4, MFC/MIC ≤ 4) or bacteriostatic/fungistatic (MBC/MIC > 4, MFC/MIC > 4) effect of the tested compounds.

3. Synthesis and NMR spectra

Typical procedure for the synthesis of *(Z)*-2-(5-benzylidene-4-oxo-2-thioxothiazolidin-3-yl)acetic acid derivatives

A mixture of selected aldehyde (4-pyrrolidinobenzaldehyde (0.39 g, 2.20 mmol) – A1, 4-(1H-pyrrol-1-yl)benzaldehyde (0.38 g, 2.20 mmol) - A2 or 4-(1H-imidazol-1-yl)benzaldehyde (0.38

g, 2.20 mmol) – A3), rhodanine-3-acetic acid (0,45 g, 2.30 mmol), ammonium acetate (0,40 g, 5.20 mmol) and acetic acid (30 mL) was heated at reflux. After 4h of intensive mixing, the solid was collected by filtration and washed with water. Crude product was crystallized from acetonitrile.

***(Z)*-2-[5-(4-(pyrrolidin-1-yl)-benzylidene)-4-oxo-2-thioxo-thiazolidin-3-yl]-acetic acid (A1)**

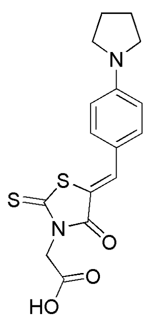

A-1 was obtained as brown solid with **54%** yield. **^{1}H NMR (400 MHz, DMSO) δ** 7.74 (s, 1H, **H**^{A8}), 7.50 (d, *J* = 9.0 Hz, 2H, **H**^{B3,B5}), 6.71 (d, *J* = 9.0 Hz, 2H, **H**^{B2,B6}), 4.71 (s, 2H, **H**^{A2}), 3.42 – 3.30 (m, 4H, **H**^{C2,C5}), 2.01 – 1.96 (m, 4H, **H**^{C3,C4}). **^{13}C NMR (100 MHz, DMSO) δ** 192.48 (**C**^{A7}), 167.43 (**C**^{A1}), 166.37 (**C**^{A4}), 149.64 (**C**^{B4}), 135.62 (**C**^{A8}), 133.58 (**C**^{B3,B5}), 119.39 (**C**^{B1}), 112.55 (**C**^{B2,B6}), 47.41 (**C**^{C2,C5}), 44.89 (**C**^{A2}), 24.88 (**C**^{C3,C4}).

***(Z)*-2-[5-(4-(1*H*-pyrrol-1-yl)-benzylidene)-4-oxo-2-thioxo-thiazolidin-3-yl]-acetic acid (A2)**

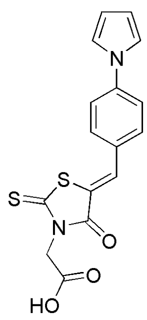

A-2 was obtained as orange solid with **63%** yield. **^{1}H NMR (400 MHz, DMSO) δ** 7.92 (s, 1H, **H**^{A8}), 7.81 (d, *J* = 8.7 Hz, 2H, **H**^{B2,B6}), 7.75 (d, *J* = 8.7 Hz, 2H, **H**^{B3,B5}), 7.53 (s, 2H, **H**^{C2,C5}), 6.33 (s, 2H, **H**^{C3,C4}), 4.75 (s, 2H, **H**^{A2}). **^{13}C NMR (100 MHz, DMSO) δ** 192.95 (**C**^{A7}), 167.27 (**C**^{A1}), 166.35 (**C**^{A4}), 141.40 (**C**^{B4}), 133.29 (**C**^{A8}), 132.56 (**C**^{B3,B5}), 129.28 (**C**^{A5}), 120.53 (**C**^{B1}), 119.40 (**C**^{B2,B6}), 119.01 (**C**^{C2,C5}), 111.57 (**C**^{C3,C4}), 45.01 (**C**^{A2})

***(Z)*-2-[5-(4-(1*H*-imidazol-1-yl)-benzylidene)-4-oxo-2-thioxo-thiazolidin-3-yl]-acetic acid (A3)**

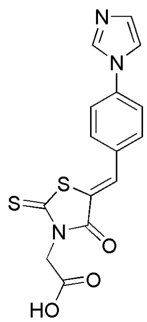

A-3 was obtained as yellow solid with **71%** yield. **^{1}H NMR (400 MHz, DMSO) δ** 8.44 (s, 1H, **H**^{C5}), 7.95 (s, 1H, **H**^{A8}), 7.92 – 7.86 (m, 3H, **H**^{C2,B2,B6}), 7.82 (d, *J* = 8.6 Hz, 2H, **H**^{B3,B5}), 7.16 (s, 1H, **H**^{C3}), 4.75 (s, 2H, **H**^{A2}). **^{13}C NMR (100 MHz, DMSO) δ** 192.99 (**C**^{A7}), 167.24 (**C**^{A1}), 166.33 (**C**^{A4}), 138.35 (**C**^{B4}), 135.68 (**C**^{C5}), 132.85 (**C**^{A8}), 132.45 (**C**^{B3,B5}), 131.02 (**C**^{A5}), 130.31 (**C**^{C3}), 121.78 (**C**^{B1}), 120.66 (**C**^{B2,B6}), 117.73 (**C**^{C2}), 45.07 (**C**^{A2}).

NMR Spectra

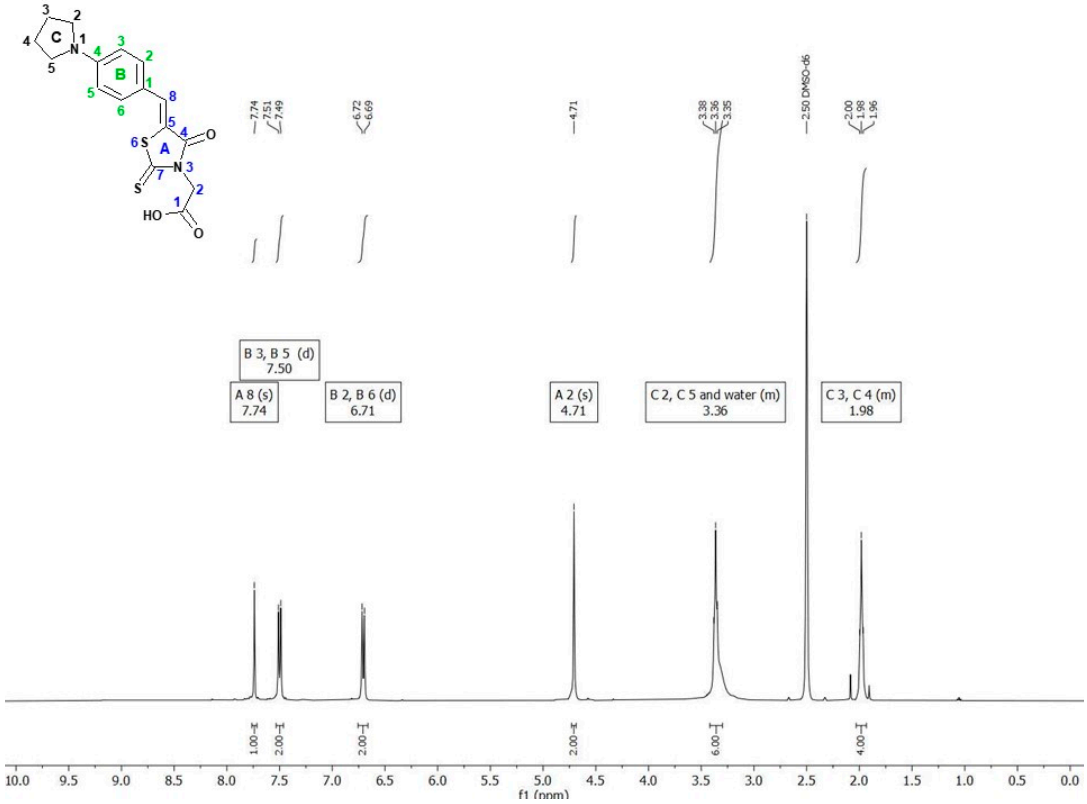

^{1}H NMR of A-1

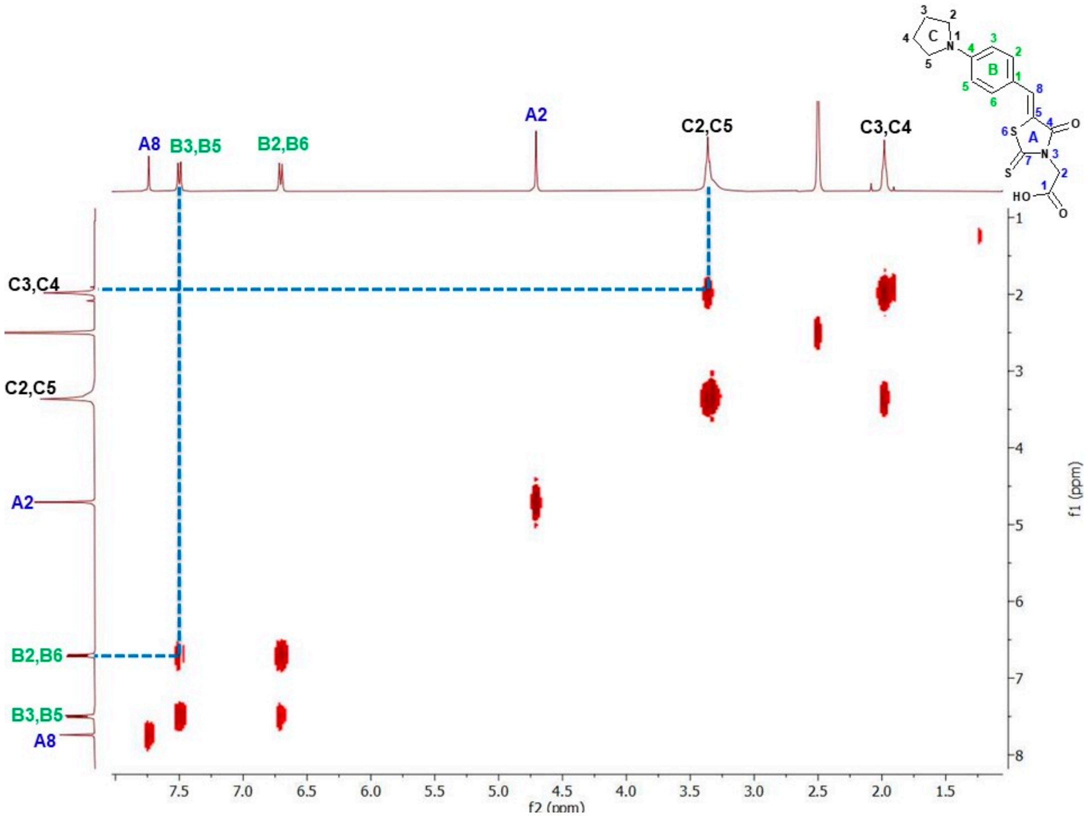

H-H COSY of A-1

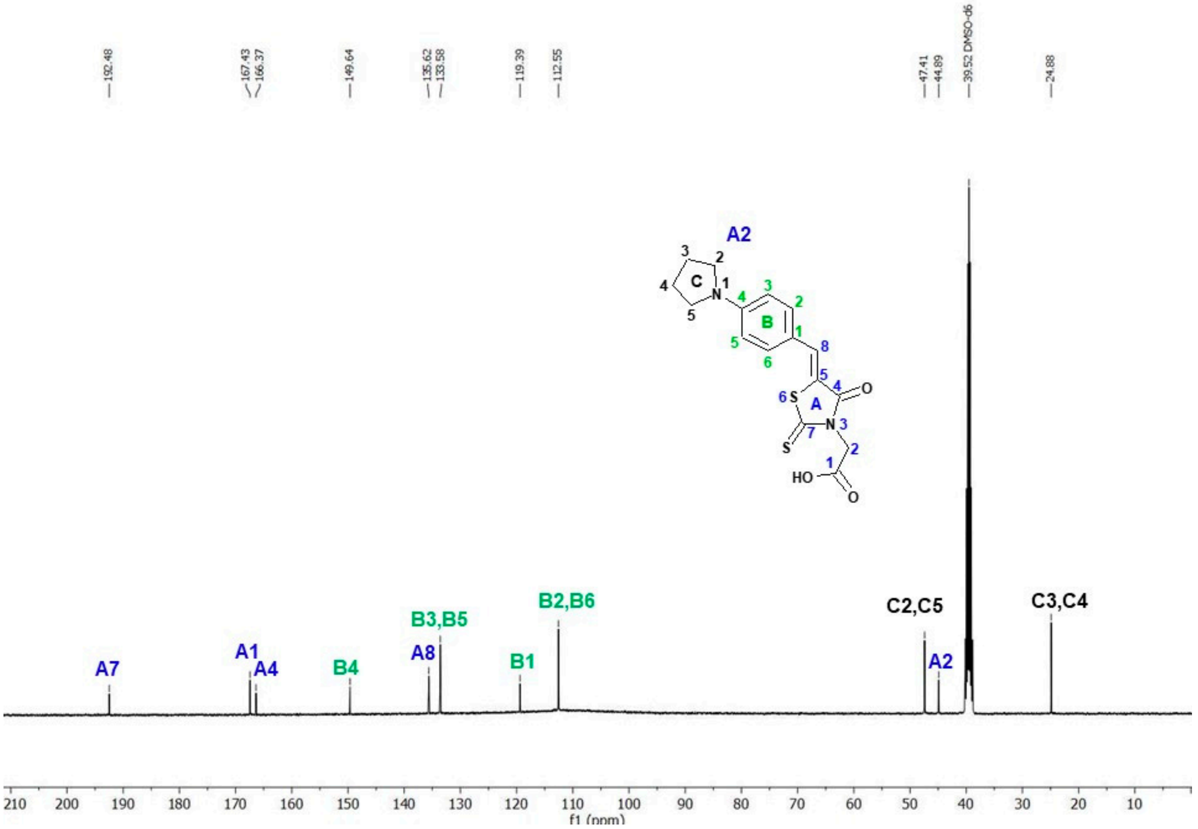

^{13}C NMR of A-1

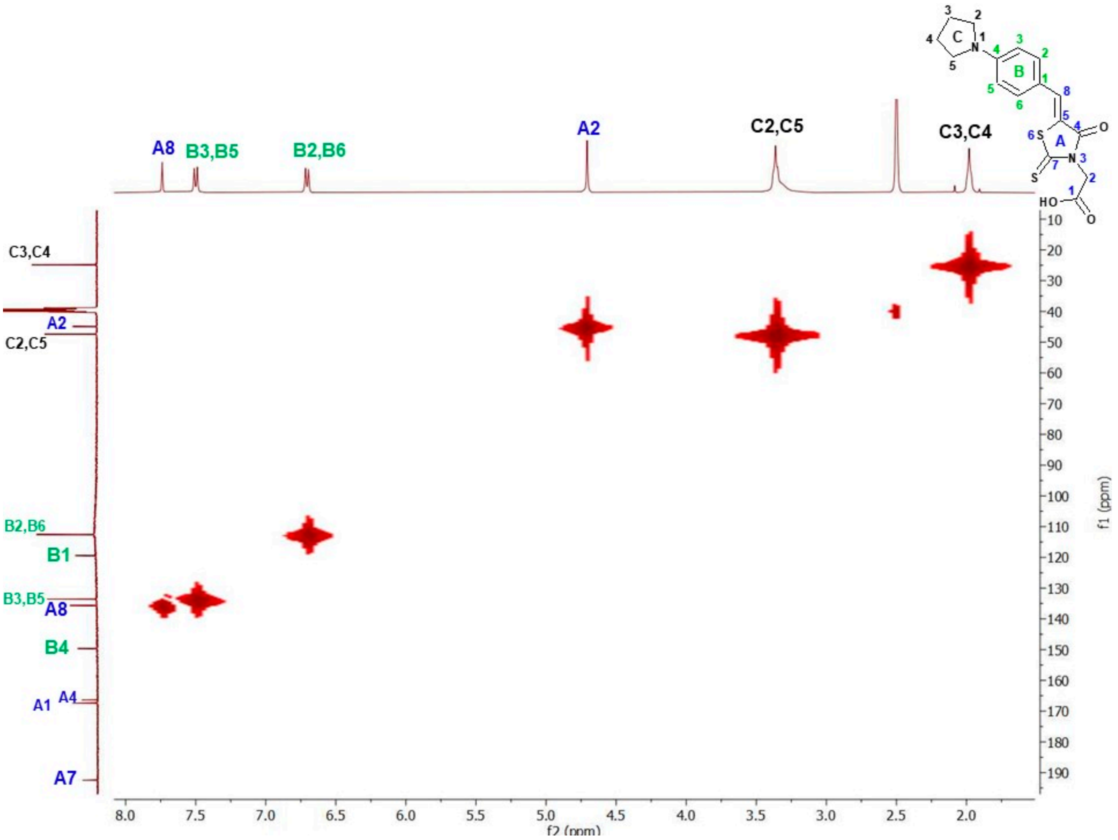

H-C HMQC of A-1

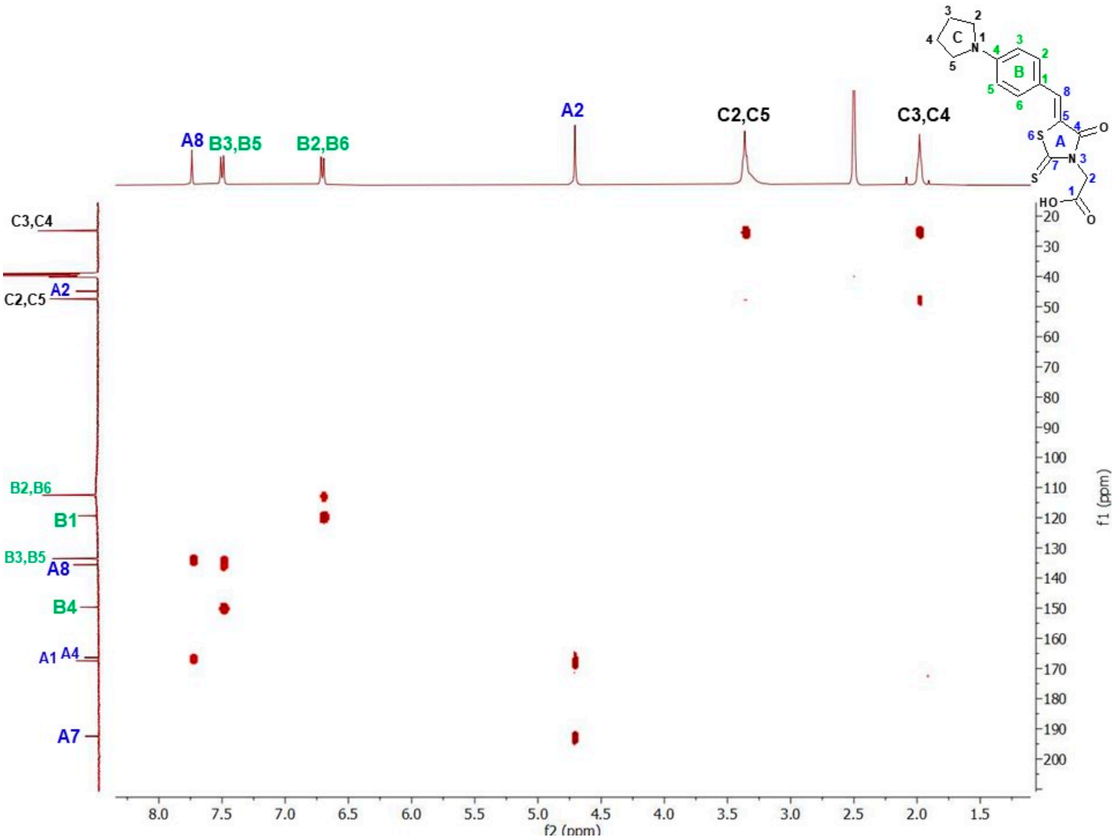

H-C HMBC of A-1

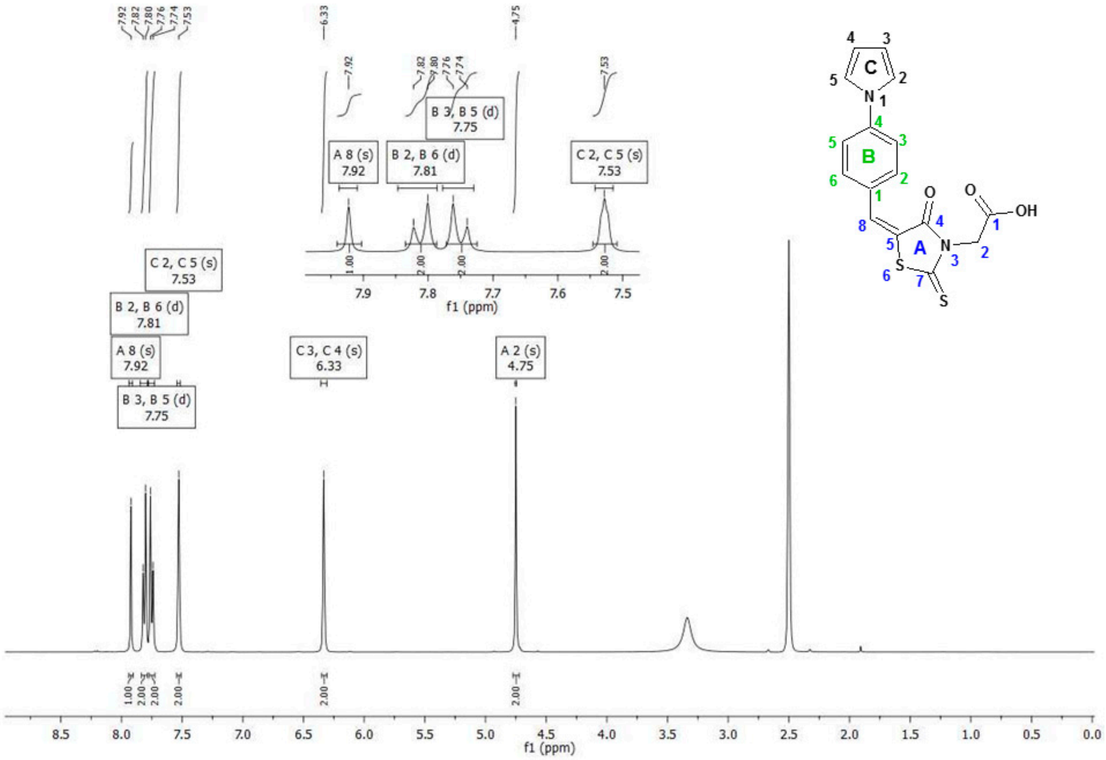

^{1}H NMR of A-2

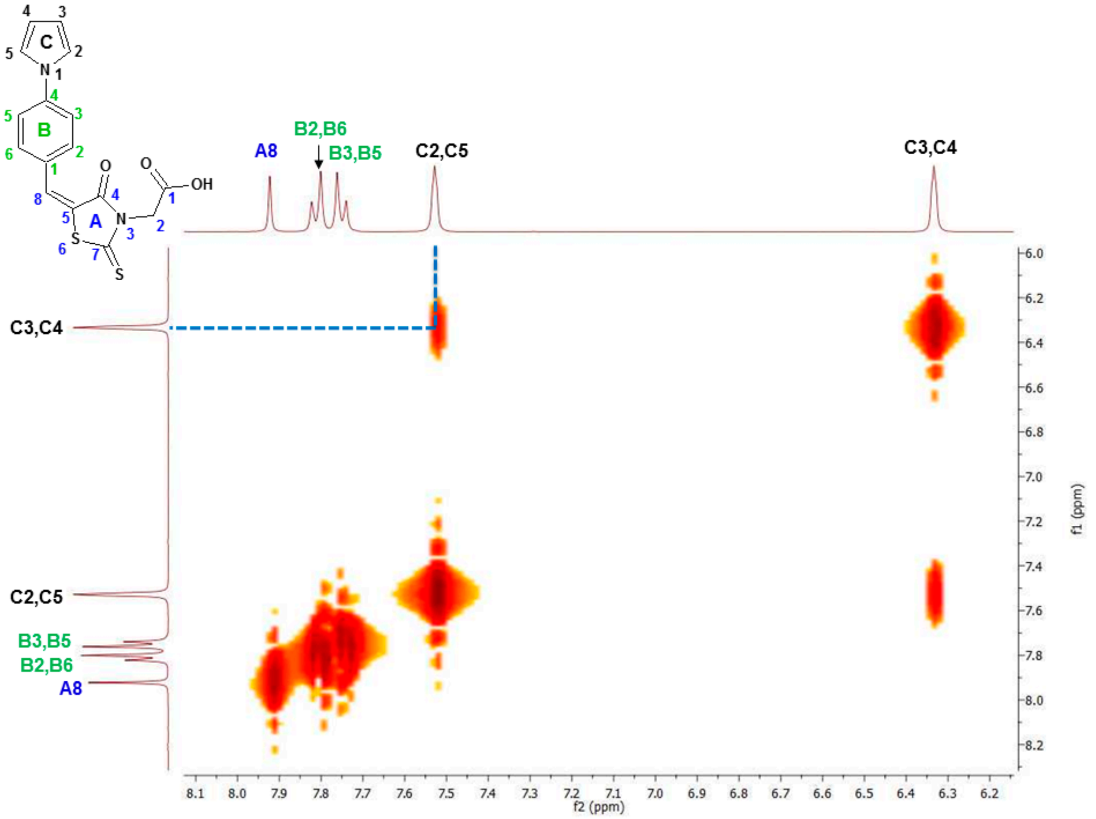

H-H COSY of A-2

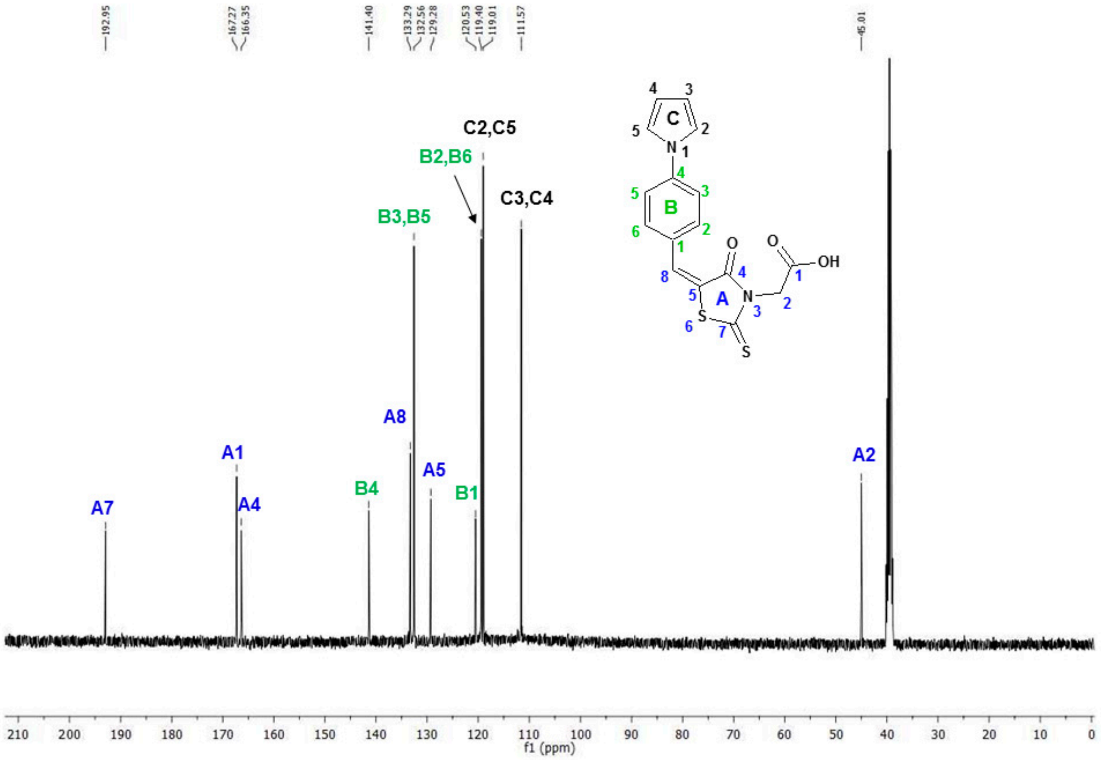

^{13}C NMR of A-2

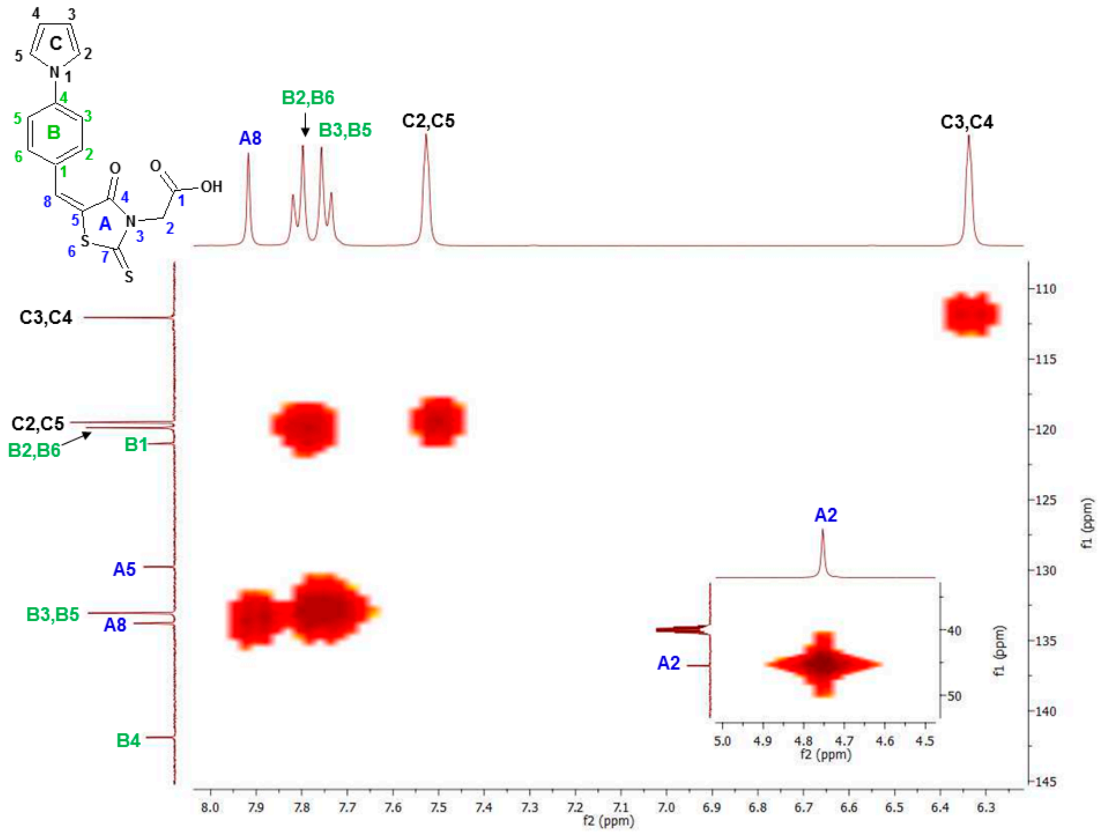

H-C HMQC of A-2

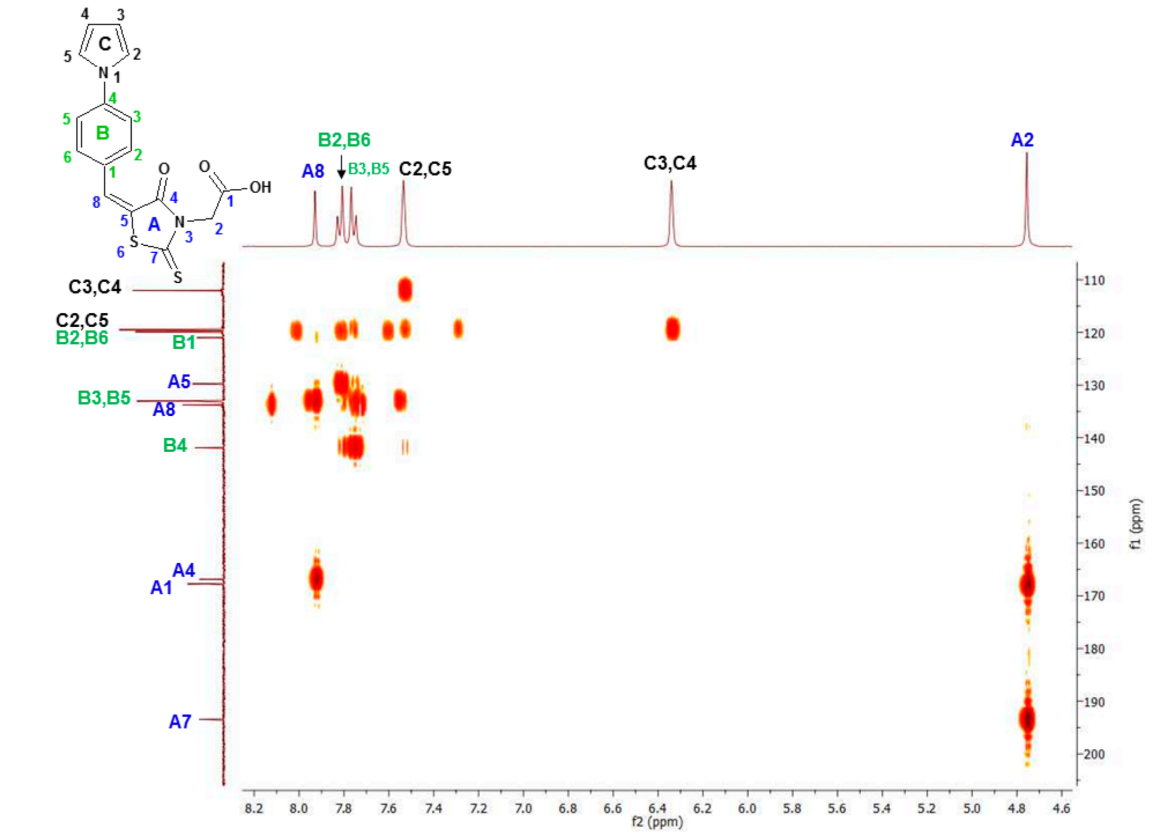

H-C HMBC of A-2

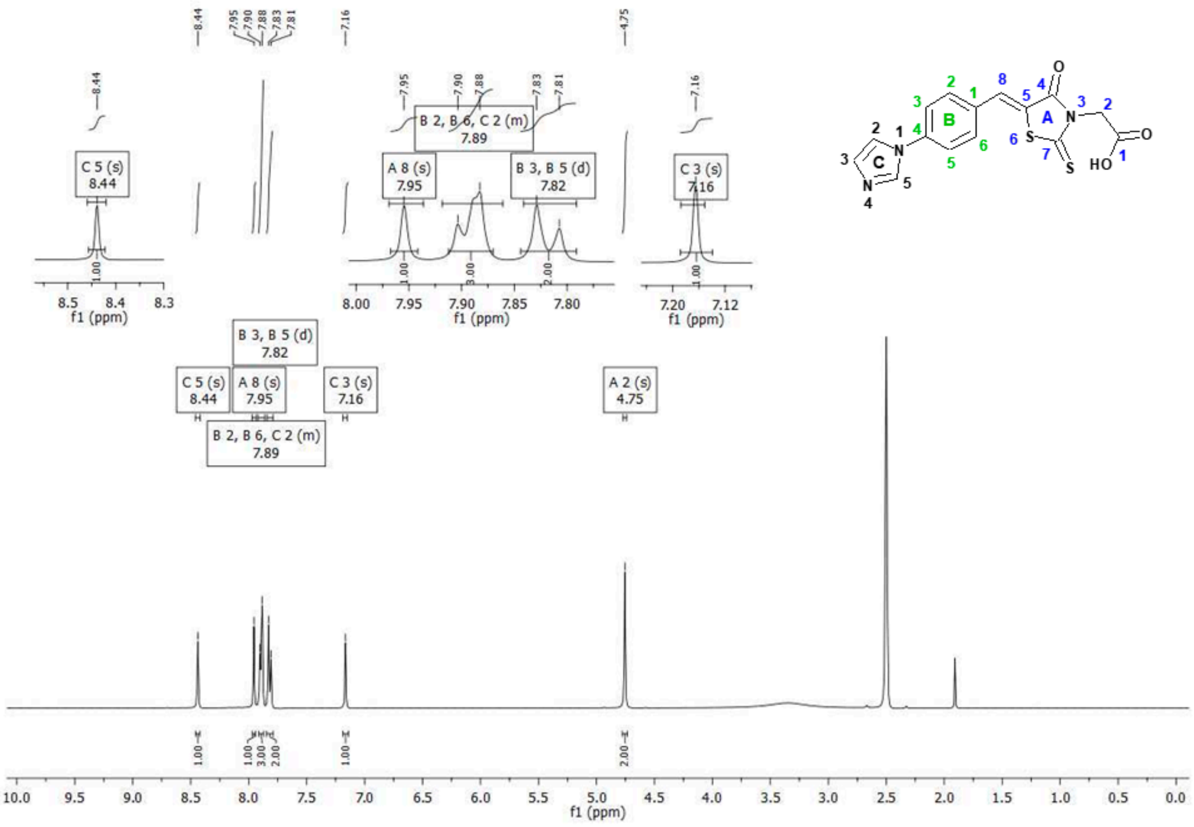

^{1}H NMR of A-3

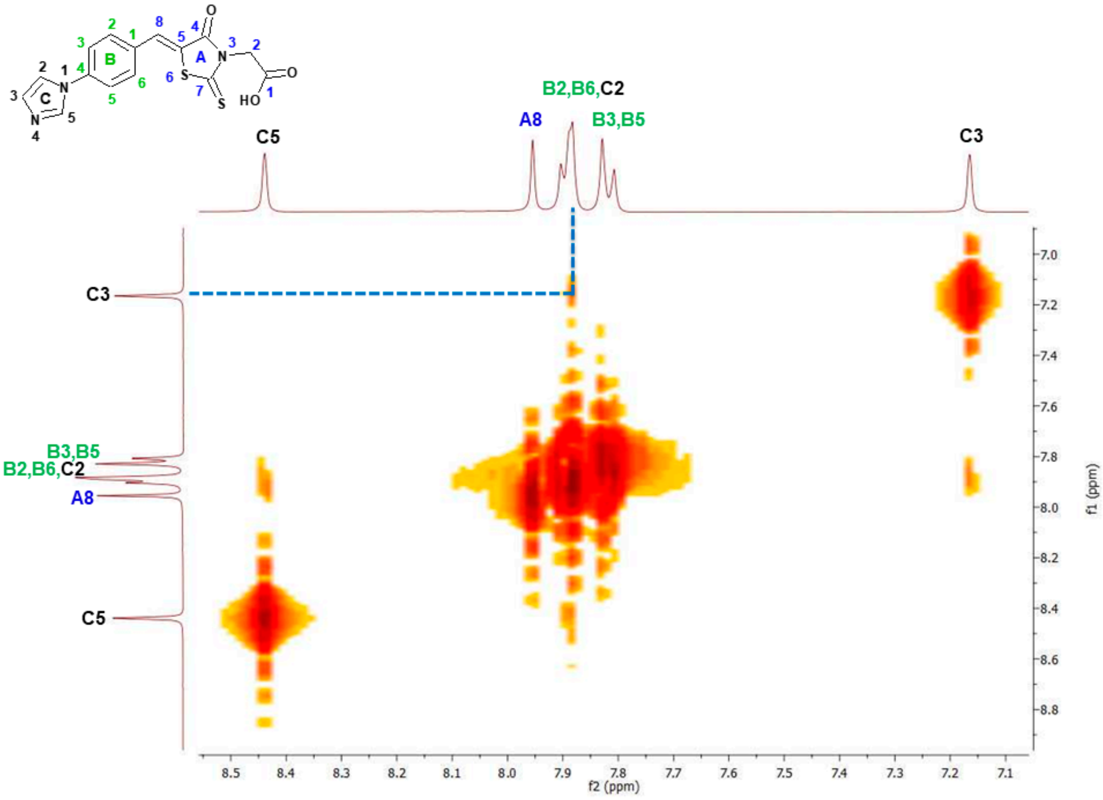

H-H COSY of A-3

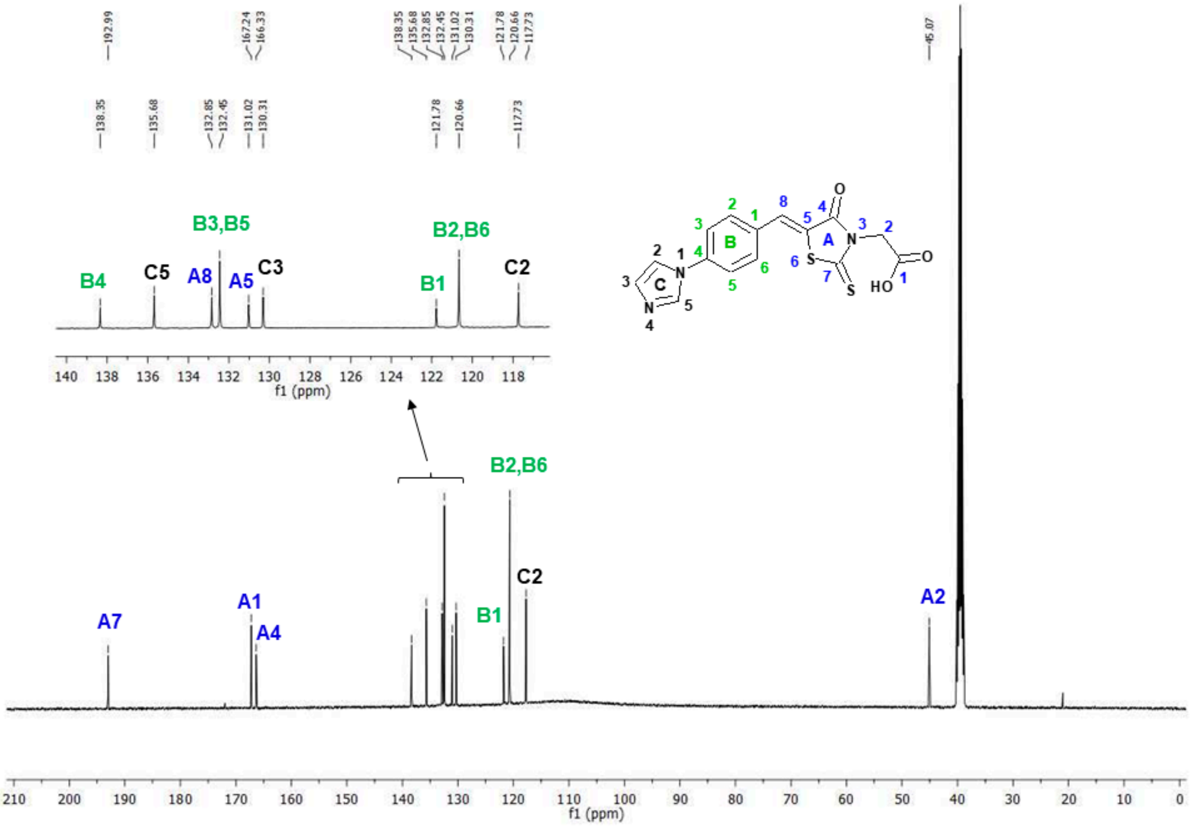

^{13}C NMR of A-3

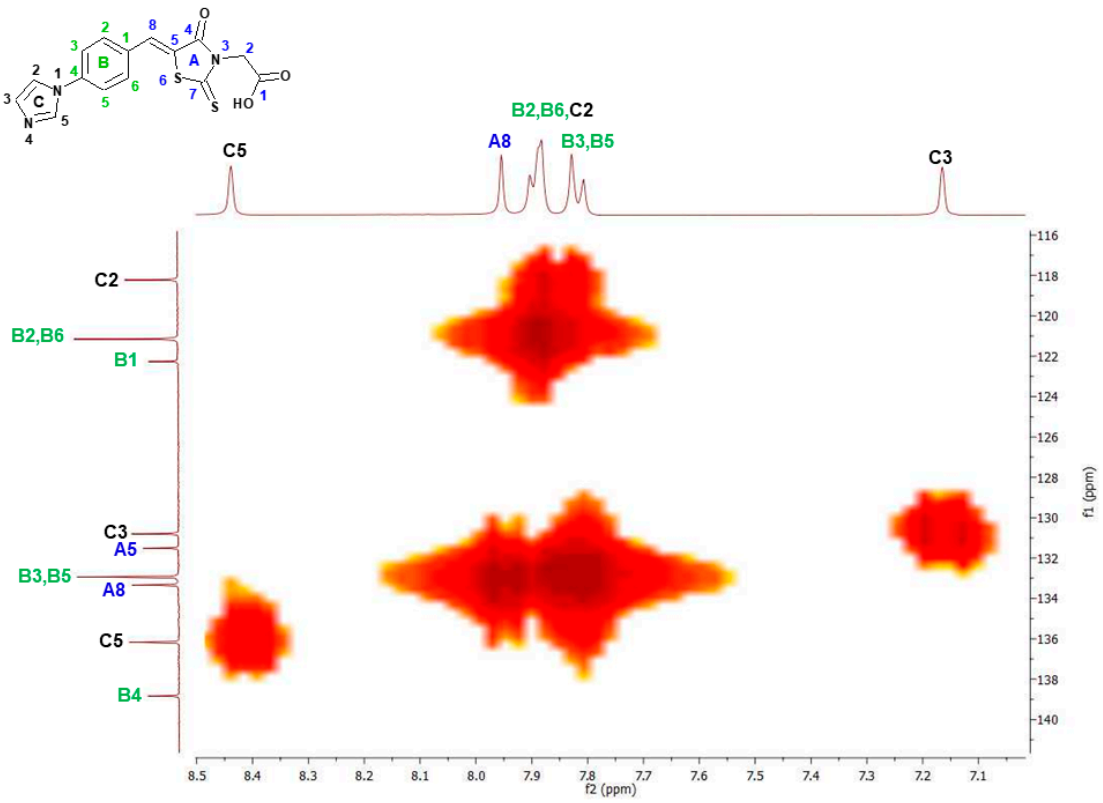

H-C HMQC of A-3

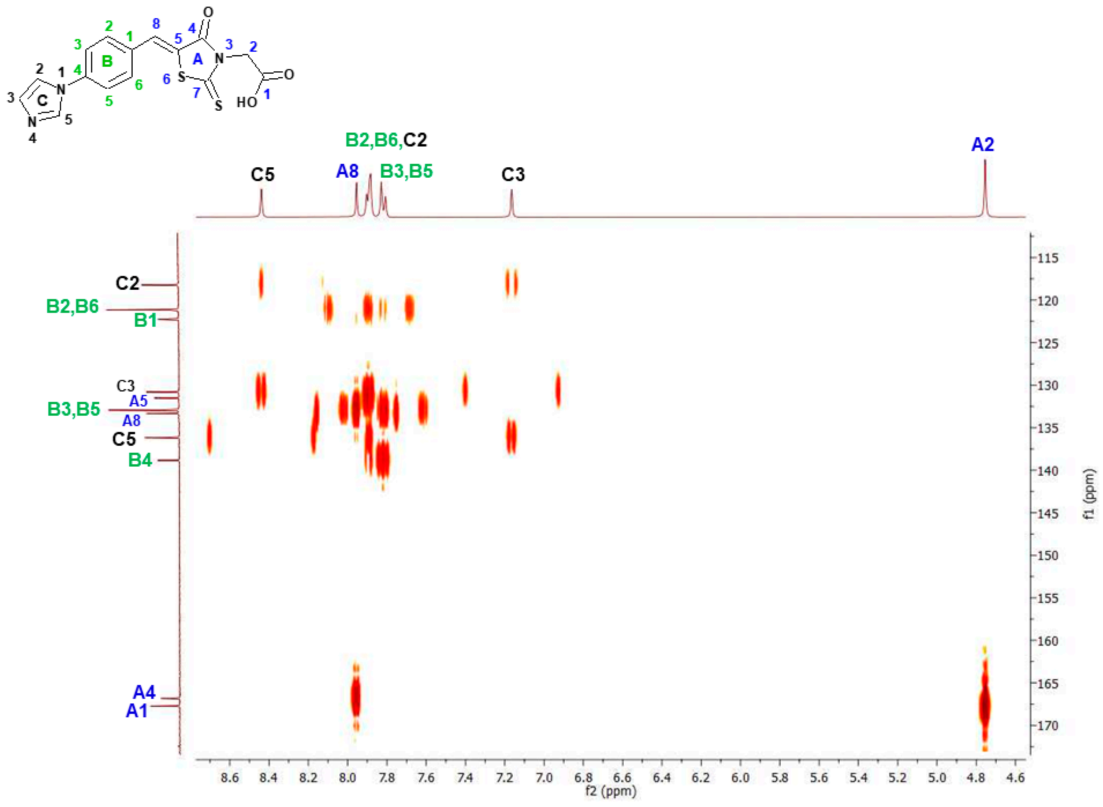

H-C HMBC of A-3

4. Thermal properties

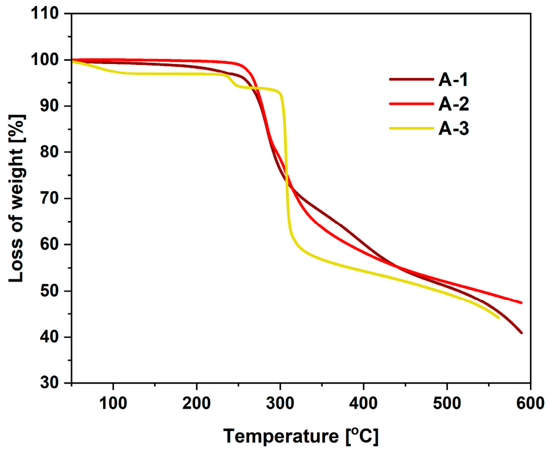

Figure S1. TGA - thermal properties of **A-1-3**

5. DFT calculations

All geometrical parameters of investigated molecules in their ground (S_{GS}) and excited (S_{CT}) states were calculated using density functional theory (DFT) approach implemented in Gaussian 09 program package [7] with TIGHT threshold option and PBE0/6-311++G(d,p) basis set. In order to verify that all the structures correspond to the minima on the potential energy surface, an analysis of Hessians was performed. The electronic properties were characterized by computations of the vertical absorption, which were obtained using the time-dependent density functional theory (TDDFT/PBE0) [8] and by including the state-specific (SS) corrected linear response (cLR) approach [9]. All spectroscopic calculations were performed using several different functionals, namely standard-hybrid PBE0 and B3LYP [10,11] functionals, as well as long-range asymptotically corrected functionals such as CAM-B3LYP [12], HSEH1PBE [13,14] and LC-ωPBE [15-17]. The dipole moments and polarities of the charge-transfer state (CT) were evaluated by numerical differentiation of the excitation energies (E) in the presence of an electric field F of 0.001 a.u. strength:

$$\Delta\mu^{i}_{CT-GS} = \frac{E_{CT}(+F^{i})-E_{CT}(-F^{i})}{-2F^{i}} - \frac{E_{GS}(+F^{i})-E_{GS}(-F^{i})}{-2F^{i}} \qquad \text{Equation (S1)}$$

where i stands for the Cartesian component of the dipole moment difference.

The isotropic average polarizability ($\langle\alpha\rangle$) and first-order hyperpolarizability (β_{vec}) were determined based on the Gaussian 09 program and defined as:

$$\langle\alpha\rangle = \frac{\alpha_{xx}+\alpha_{yy}+\alpha_{zz}}{3} \qquad \text{Equation (S2)}$$

$$\beta_{vec} = \sum_{i=x,y,z}\frac{\mu_i\beta_i}{|\mu|} \qquad \text{Equation (S3)}$$

where $\beta_i (i = x, y, z)$ is given by $\beta_i = \left(\frac{1}{3}\right) \sum_{j=x,y,z} (\beta_{ijj} + \beta_{jij} + \beta_{jji})$

The density differences were obtained at the PBE0/6-311++G(d,p) level and are represented with a contour threshold of 0.02 a.u. In these graphs, the blue (purple) zones indicate density decrease (increase) upon electronic transition. The charge transfer parameters, namely the charge-transfer distance (D_{CT}) and the amount of transferred charge (q_{CT}), have been determined following a Le Bahers' procedure [18]. The solvent effect on the linear and nonlinear optical properties has been taken into account using the Integral Equation Formalism for the Polarizable Continuum Model (IEF–PCM) [19,20].

The binding properties of considered dyes were studied by performing a series of AutoDock 4.2 [21-23] simulations. The starting structures of each complex comprised Human Serum Albuminum (HAS) taken from PDB ID [24] and each dye as a ligand. The docking region on the target protein was defined by establishing a cubic grid box with the dimensions of 16Å and a grid spacing of 1Å. In AutoDock 4.2 simulations, the Lamarkian genetic algorithm was employed to identify the appropriate binding energy and conformation of compounds. For each atom of the receptor molecule, Gasteiger charges were calculated. The investigation of the binding site was performed using a united-atom scoring function implemented in AutoDock Vina [25]. The docking procedure was repeated ten times for each lysine and this enabled for identification of the sites with the highest affinity of fluorescent probes.

The biological activities were simulated using a combination of the 3D/4D QSAR BiS/MC and CoCon algorithms [26-28].

Table S1. The frontier orbital energies in tested solvents for ***Z***-isomers. Values are given in [eV].

	E_{HOMO}	E_{LUMO}	E_{GAP}	η	μ	χ	σ	S	ω
**A1**									
Toluene	-5.7598	-2.4873	3.2725	1.6362	-4.1236	4.1236	0.6112	0.8181	5.1961
$CHCl_3$	-5.7519	-2.5442	3.2077	1.6038	-4.1481	4.1481	0.6235	0.8019	5.3641
THF	-5.7497	-2.5701	3.1797	1.5898	-4.1599	4.1599	0.6290	0.7949	5.4423
MeOH	-5.7478	-2.6101	3.1378	1.5689	-4.1790	4.1790	0.6374	0.7844	5.5657
MeCN	-5.7478	-2.6114	3.1364	1.5682	-4.1796	4.1796	0.6377	0.7841	5.5699
DMSO	-5.7478	-2.6120	3.1359	1.5679	-4.1799	4.1799	0.6378	0.7840	5.5716
**A2**									
Toluene	-6.4973	-2.9029	3.5944	1.7972	-4.7001	4.7001	0.5564	0.8986	6.1459
$CHCl_3$	-6.4832	-2.9070	3.5762	1.7881	-4.6951	4.6951	0.5593	0.8940	6.1640

THF	-6.4785	-2.9100	3.5686	1.7843	-4.6943	4.6943	0.5605	0.8921	6.1751
MeOH	-6.4695	-2.9122	3.5574	1.7787	-4.6909	4.6909	0.5622	0.8893	6.1854
MeCN	-6.4723	-2.9149	3.5574	1.7787	-4.6936	4.6936	0.5622	0.8893	6.1926
DMSO	-6.4723	-2.9149	3.5574	1.7787	-4.6936	4.6936	0.5622	0.8893	6.1926
**A3**									
Toluene	-6.7240	-3.0115	3.7125	1.8563	-4.8677	4.8677	0.5387	0.9281	6.3824
$CHCl_3$	-6.6960	-2.9911	3.7049	1.8524	-4.8435	4.8435	0.5398	0.9262	6.3321
THF	-6.6845	-2.9832	3.7014	1.8507	-4.8339	4.8339	0.5403	0.9253	6.3129
MeOH	-6.6671	-2.9718	3.6954	1.8477	-4.8194	4.8194	0.5412	0.9238	6.2854
MeCN	-6.6663	-2.9715	3.6948	1.8474	-4.8189	4.8189	0.5413	0.9237	6.2849
DMSO	-6.6663	-2.9715	3.6948	1.8474	-4.8189	4.8189	0.5413	0.9237	6.2849

Table S2. CT parameters for the bright low-lying excited state. Values q_{CT} are given in [e] and D_{CT} in [Å].

	A1		A2		A3	
	q_{CT}	D_{CT}	q_{CT}	D_{CT}	q_{CT}	D_{CT}
Toluene	0.518	2.919	0.525	2.956	0.845	1.921
$CHCl_3$	0.521	3.186	0.540	3.210	0.842	2.117
THF	0.521	3.286	0.542	3.373	0.840	2.212
MeOH	0.521	3.439	0.545	3.561	0.839	2.367
MeCN	0.521	3.439	0.547	3.557	0.839	2.370
DMSO	0.524	3.441	0.549	3.545	0.839	2.380

Table S3. Free energies (ΔG_{solv}, kcal/mol) of solvation.

	**A1**	**A2**	**A3**
Toluene	-17.99	-16.20	-19.74
$CHCl_3$	-19.12	-19.63	-23.62
THF	-20.37	-19.60	-25.06
MeOH	-22.48	-19.04	-22.90
MeCN	-22.30	-19.12	-25.78
DMSO	-19.91	-19.10	-22.81

Table S4. The vertical excitation energies (in nm).

	B3LYP		CAM-B3LYP		HSEH1PBE		LC-ωPBE		PBE0	
	λ	f	λ	f	λ	f	λ	f	λ	f
**A1**										
Toluene	458.41	1.1180	407.90	1.3172	454.16	1.1290	378.49	1.4163	461.05	1.1781

$CHCl_3$	464.93	1.1069	413.89	1.3164	460.69	1.1175	384.43	1.4183	468.33	1.1694
THF	466.94	1.0967	415.85	1.3112	462.70	1.1071	386.48	1.4144	465.25	1.1605
MeOH	469.02	1.0752	418.00	1.2978	464.78	1.0849	388.89	1.4034	471.22	1.1406
MeCN	469.87	1.0802	418.77	1.3023	465.62	1.0900	389.60	1.4078	473.06	1.1456
DMSO	473.37	1.1008	421.77	1.3211	469.07	1.1111	392.24	1.4259	479.45	1.1661
**A2**										
Toluene	429.04	1.1234	382.79	1.2827	424.21	0.1193	368.82	1.3585	410.33	1.1570
$CHCl_3$	425.39	1.0642	378.78	1.2709	426.66	1.1178	364.73	1.3483	405.47	1.1616
THF	425.25	1.0994	376.79	1.2589	427.44	1.1094	362.70	1.3389	402.30	1.1591
MeOH	424.52	1.0765	374.01	1.1594	427.95	1.0836	359.76	1.3150	399.48	1.1348
MeCN	425.01	1.0872	373.44	1.1132	428.31	1.0920	359.22	1.3233	397.96	1.1444
DMSO	427.26	1.1073	374.77	1.2475	428.34	1.1125	359.10	1.3410	402.11	1.1642
**A3**										
Toluene	409.49	0.9979	362.86	1.1939	405.97	0.9303	334.52	1.2698	395.71	1.0800
$CHCl_3$	409.33	0.9957	363.16	1.1821	405.67	0.9324	334.84	1.2572	375.60	1.0718
THF	408.81	0.9891	362.90	1.1726	405.13	0.9266	334.64	1.2474	388.11	1.0633
MeOH	407.54	0.9706	362.04	1.1533	403.86	0.9094	333.94	1.2276	383.88	1.0447
MeCN	407.99	0.9764	362.47	1.1574	404.26	0.9170	334.31	1.2316	378.33	1.0497
DMSO	409.94	1.0016	364.24	1.1751	406.01	0.9479	335.84	1.2491	389.25	1.0709

Table S5. The cLR corrected excitation energies (in nm).

	**A1**	**A2**	**A3**
Toluene	457.93	425.04	398.2
$CHCl_3$	465.62	412.71	386.89
THF	468.73	410.76	394.66
MeOH	473.07	407.34	390.17
MeCN	481.44	407.14	390.66
DMSO	486.80	407.68	392.95

Table S6. Calculated values of dipole moments (in D) for the ground and CT excited state.

	**A1**		**A2**		**A3**	
	μ_{GS}	μ_{CT}	μ_{GS}	μ_{CT}	μ_{GS}	μ_{CT}
Toluene	13.29	20.61	5.66	8.29	2.10	4.40
$CHCl_3$	14.53	22.46	6.16	11.43	2.42	4.44

THF	15.09	23.10	6.39	13.22	2.58	4.45
MeOH	15.96	24.53	5.56	14.81	2.86	4.51
MeCN	15.99	24.57	6.76	15.19	2.87	4.56
DMSO	16.04	24.66	6.78	15.31	2.88	5.05

Table S7. Nonlinear properties of tested isomers. Values are given in (a.u.).

	A1		A2		A3	
	$\langle\alpha\rangle$	β	$\langle\alpha\rangle$	β	$\langle\alpha\rangle$	β
Toluene	385.46	10727.20	363.34	7709.07	344.59	4107.50
$CHCl_3$	424.49	14650.21	394.44	9684.80	372.90	4890.27
THF	443.38	16724.54	409.08	10605.80	386.30	5227.10
MeOH	474.70	20390.60	432.69	12197.77	408.13	5712.17
MeCN	475.65	20499.02	433.59	12089.51	408.76	5723.49
DMSO	477.90	20797.62	435.29	12187.90	410.34	5759.18

Figure S2. HOMO / LUMO plots.

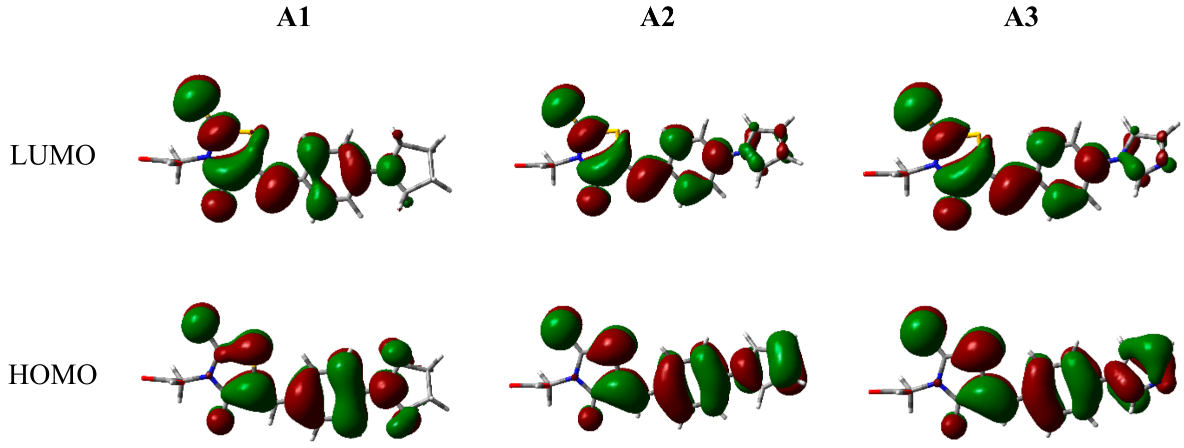

Figure S3. The MEP surfaces.

A1 **A2** **A3**

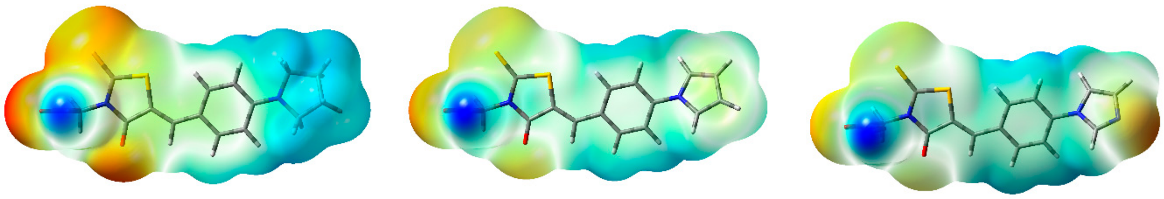

Figure S4. Density difference plots.

A1 **A2** **A3**

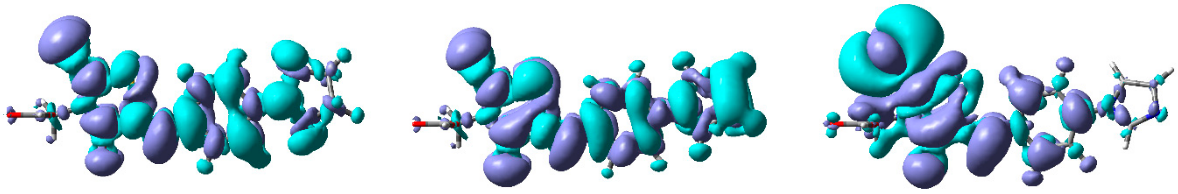

Figure S5. Structural changes of the tested derivatives after spatial adjustment to the protein

A1 **A2** **A3**

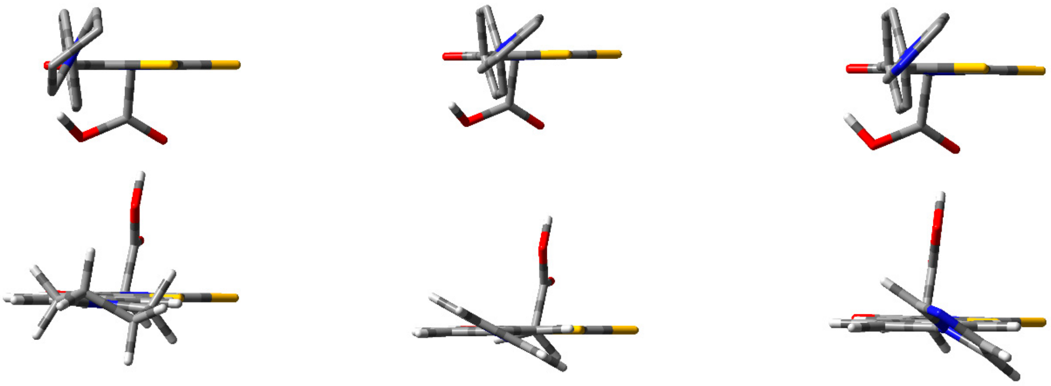

6. Optical properties

Table S8. Absorption and emission of **A-1-3**

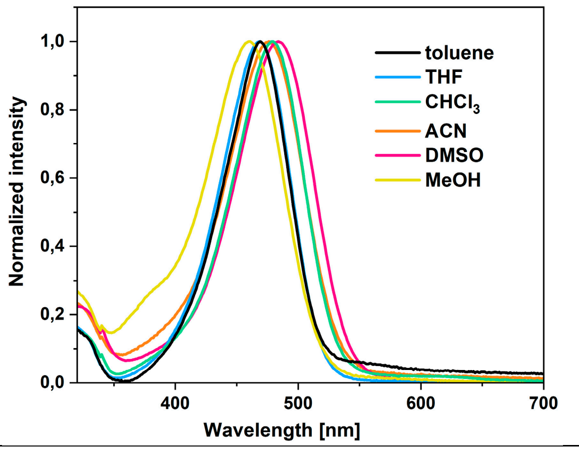

Absorption of A-1

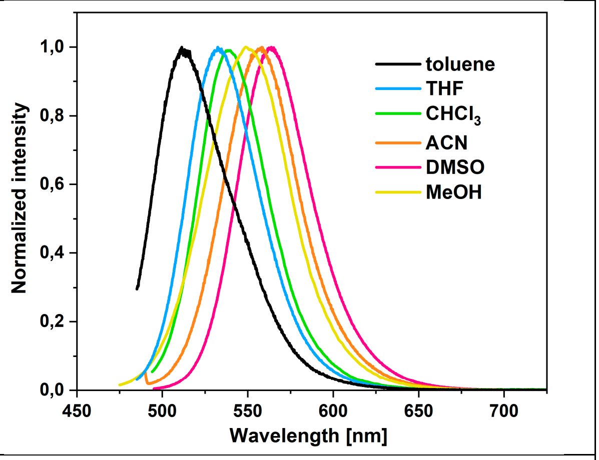

Emission of A-1

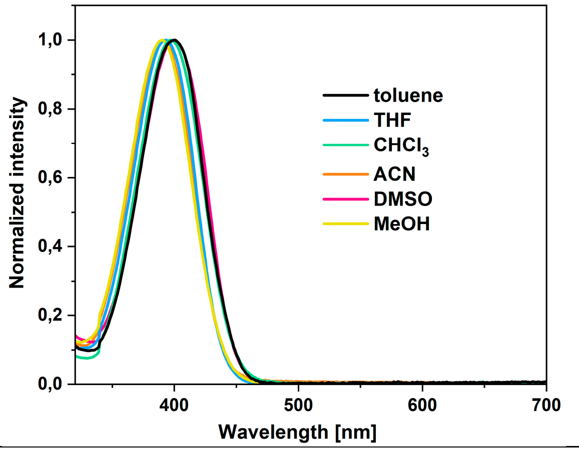

Absorption of A-2

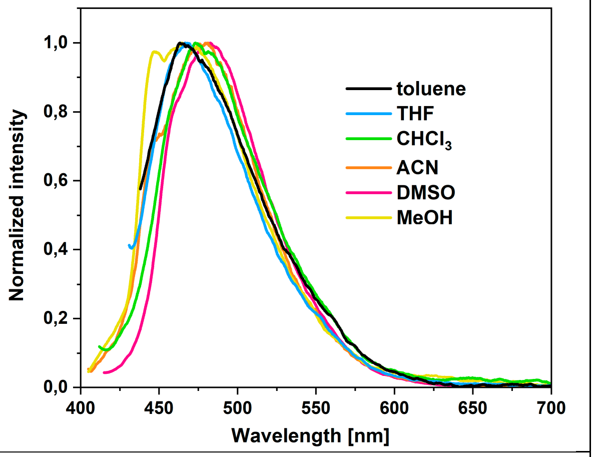

Emission of A-2

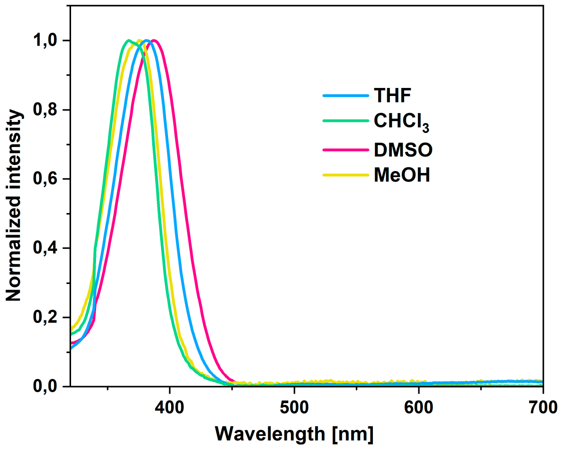

Absorption of A-3

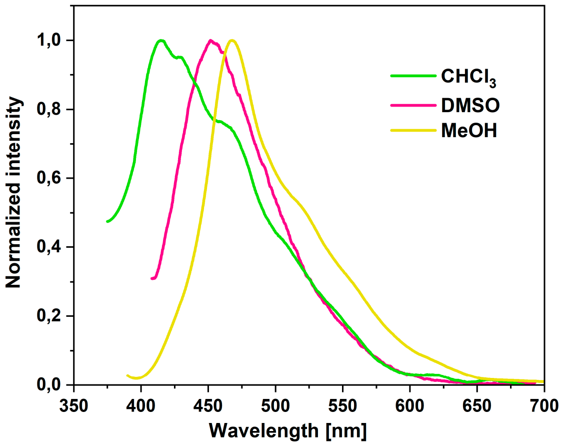

Emission of A-3

Table S9. UV-Vis spectra in DMSO recorded at time intervals at room temperature

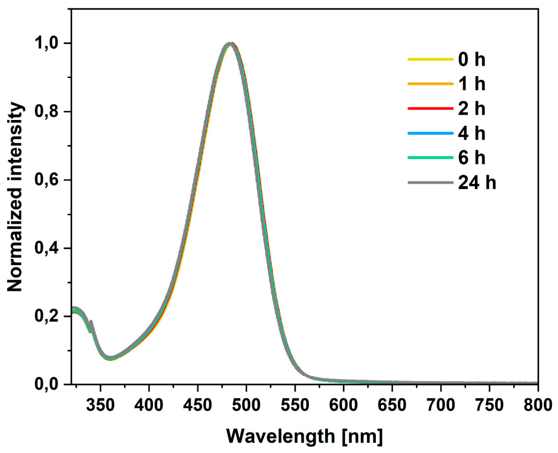

A-1

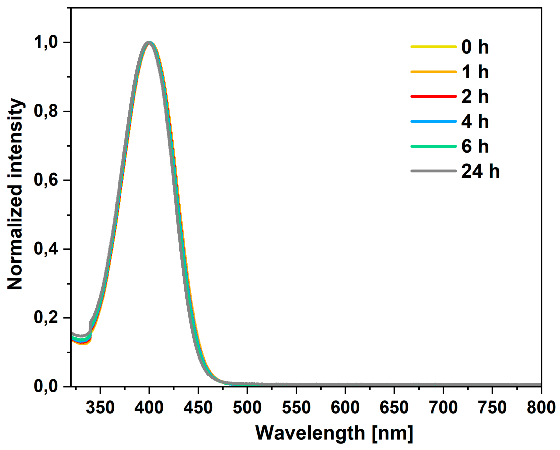

A-2

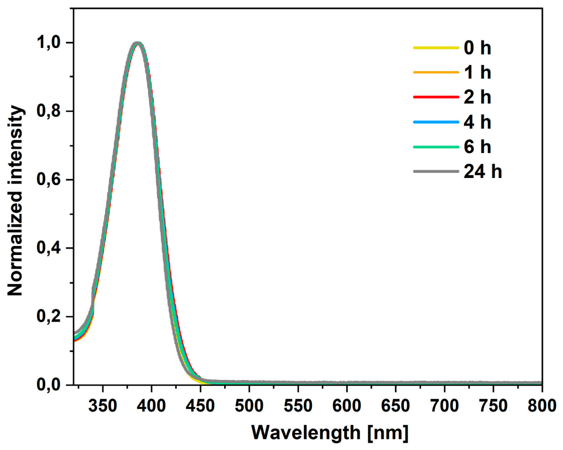

A-3

Table S10. The Lippert-Mataga plots

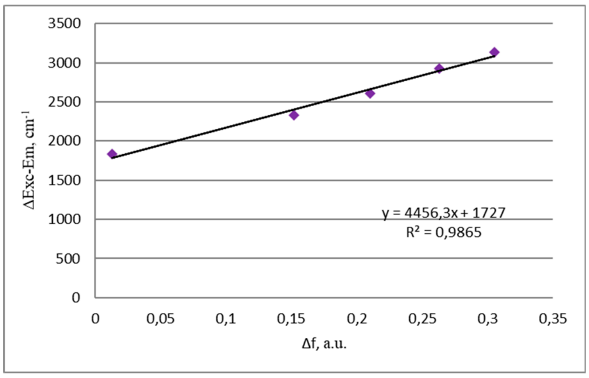

A-1

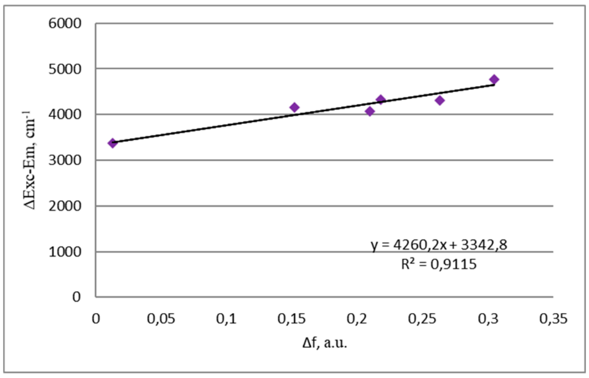

A-2

7. Literature

[1] A. Brouwer. *Pure Appl. Chem.*, 2011, 83, 2213–2228.

[2] European Committee for Antimicrobial Susceptibility Testing (EUCAST) (2003) determination of minimum inhibitory concentrations (MICs) of antibacterial agents by broth dilution. EUCAST discussion document E. Dis 5.1, *Clin. Microbiol. Infect.*, 2003, 9, 1-7.

[3] Clinical and Laboratory Standards Institute. Reference method for broth dilution antifungal susceptibility testing of yeasts. M27-S4. Clinical and Laboratory Standards Institute, Wayne, PA, USA, 2012.

[4] A. Biernasiuk, M. Kawczyńska, A. Berecka-Rycerz, B. Rosada, A. Gumieniczek, A. Malm, K. Dzitko, K.Z. Łączkowski. *Med. Chem. Res.*, 2019, 28, 2023-2036.

[5] I. Wiegand, K. Hilpert, R.E.W Hancock. *Nat. Protoc.*, 2008, 3, 163-75.

[6] F. O'Donnell, T.J. Smyth, V.N. Ramachandran, W.F. Smyth. *Int. J. Antimicrob. Agents.*, 2010, 35, 30-38.

[7] M.J. Frisch, G.W. Trucks, G.B. Schlegel et al. Gaussian 09, Revision A.1, Gaussian, Inc., Wallingford CT, 2009

[8] C. Adamo, G.E. Scuseria, V. Barone, *J. Chem. Phys.* 1999, 111, 2889-2899. DOI: 10.1063/1.479571

[9] C. Guido, S. Caprasecca, https://www1.dcci.unipi.it/molecolab/tools/white-papers/pisalr/; 2016. DOI: 10.13140/RG.2.1.1903.7845

[10] J. P. Perdew, K. Burke, and M. Ernzerhof, *Phys. Rev. Lett.*, 1996, **77**, 3865-68. DOI: 10.1103/PhysRevLett.77.3865

[11] J. P. Perdew, K. Burke, and M. Ernzerhof, *Phys. Rev. Lett.*, 1997, **78**, 1396. DOI: 10.1103/PhysRevLett.78.1396

[12] T. Yanai, D.P. Tew, N.C. Handy, *Chem. Phys. Lett.*, 2004, **393,** 51-57. DOI:10.1016/j.cplett.2004.06.011

[13] J. Heyd, G. E. Scuseria, *J. Chem. Phys.* 2004, **120**, 7274. DOI: 10.1063/1.1668634

[14] J. Heyd, G. E. Scuseria, M. Ernzerhof , *J. Chem. Phys.,* 2006, **124**, 219906. DOI: 10.1063/1.2204597

[15] H. Iikura, T. Tsuneda T, Yanai, K. Hirao K, *J. Chem. Phys.*, 2001, **115**, 3540-3544. DOI: 10.1063/1.1383587

[16] O.A. Vydrov, G.E. Scuseria, *J. Chem. Phys.*, 2006, **125**, 234109-9. DOI: 10.1063/1.2409292.

[17] O.A. Vydrov, G.E. Scuseria GE, J.P. Perdew, *J. Chem. Phys.*, 2007, **126**, 1541009-9. DOI: 10.1063/1.2723119

[18] M. Caricato, M.. *J. Chem. Phys*., 2013, **139**, 044116. DOI: 10.1063/1.4816482

[19] T. Le Bahers, C. Adamo, I. Ciofini, *J. Chem. Theory Comput*., 2011, **7**, 2498–2506. DOI: 10.1021/ct200308m

[20] M.T. Cancés, B. Mennucci, J. Tomasi, *J. Chem. Phys*., 1997, **107**, 3032-3041. DOI: 10.1063/1.474659

[21] G.M. Morris, R. Huey, W. Lindstrom, M.F. Sanner, R.K. Belew, D.S. Goodsell, A.J. Olson, *J. Comput. Chem*. 2009, **30**, 2785-2791. DOI: 10.1002/jcc.21256

[22] S. Cosconati, S. Forli, A.L. Perryman, R. Harris, D.S. Goodsell, A.J. Olson, *Expert Opin. Drug Discovery,* 2010, **5**, 597-607. DOI: 10.1517/17460441.2010.484460

[23] S. Forli, A.J. Olson, *J. Med. Chem.* 2012, **55**, 623-638. DOI: 10.1021/jm2005145

[24] S. Sugio, S. Mochizuki, M. Noda, A. Kashima, Crystal structure of human serum albumin, Protein Data Bank, Rutgers University, 1998. https://doi.org/10.2210/pdb1ao6/pdb

[25] O. Trott, A.J. Olson, *J. Comp. Chem*. 2010, **31**, 455-461. DOI: 10.1002/jcc.21334

[26] V. Potemkin, M. Grishina, *Drug Discov. Today* 2008, **13(21-22)**, 952-959. DOI: 10.1016/j.drudis.2008.07.006

[27] V. Potemkin, M. Grishina, *J. Comput. Aided. Mol. Des*. 2008, 22(6-7), 489-505. DOI: 10.1007/s10822-008-9203-x

[28] V. Potemkin, A.A. Pogrebnoy, M.A. Grishina, *J. Chem. Inf. Model*. 2009, **49(6)**, 1389-406. DOI: 10.1021/ci800405n
